# Harnessing the potency of scorpion venom-derived proteins: applications in cancer therapy

**DOI:** 10.1186/s40643-024-00805-0

**Published:** 2024-10-03

**Authors:** Jihad El-Qassas, Mahmoud Abd El-Atti, Nagwa El-Badri

**Affiliations:** 1 Department of Zoology, Faculty of Science, Zagazig University, Zagazig, 44519 Egypt; 2 Center of Excellence for Stem Cells and Regenerative Medicine, Zewail City of Science and Technology, 6th of October City, Giza, 12578 Egypt

**Keywords:** SV-derived peptides, Anticancer agents, Cancer therapy, Drug development, Drug discovery, Peptide therapeutics

## Abstract

Despite breakthroughs in the development of cancer diagnosis and therapy, most current therapeutic approaches lack precise specificity and sensitivity, resulting in damage to healthy cells. Selective delivery of anti-cancer agents is thus an important goal of cancer therapy. Scorpion venom (SV) and/or body parts have been used since early civilizations for medicinal purposes, and in cultures, SV is still applied to the treatment of several diseases including cancer. SV contains numerous active micro and macromolecules with diverse pharmacological effects. These include potent anti-microbial, anti-viral, anti-inflammatory, and anti-cancer properties. This review focuses on the recent advances of SV-derived peptides as promising anti-cancer agents and their diagnostic and therapeutic potential applications in cancers such as glioma, breast cancer, prostate cancer, and colon cancer. Well-characterized SV-derived peptides are thus needed to serve as potent and selective adjuvant therapy for cancer, to significantly enhance the patients’ survival and wellbeing.

**Graphical abstract:**

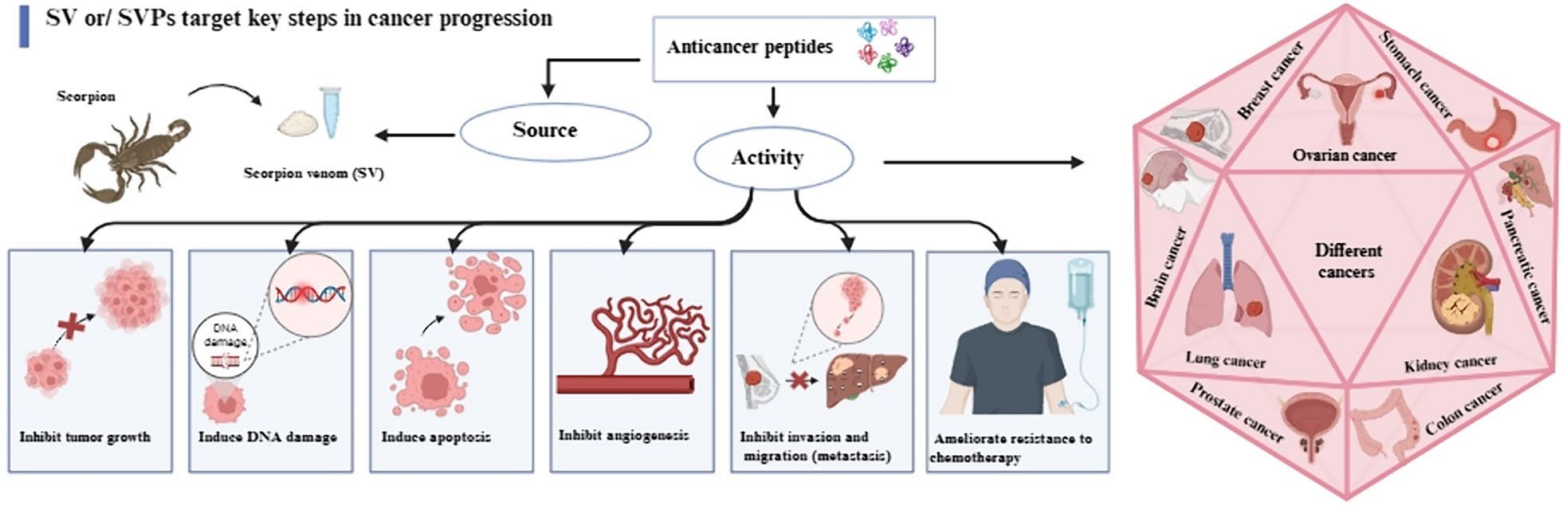

## Introduction

**Fig. 1:**
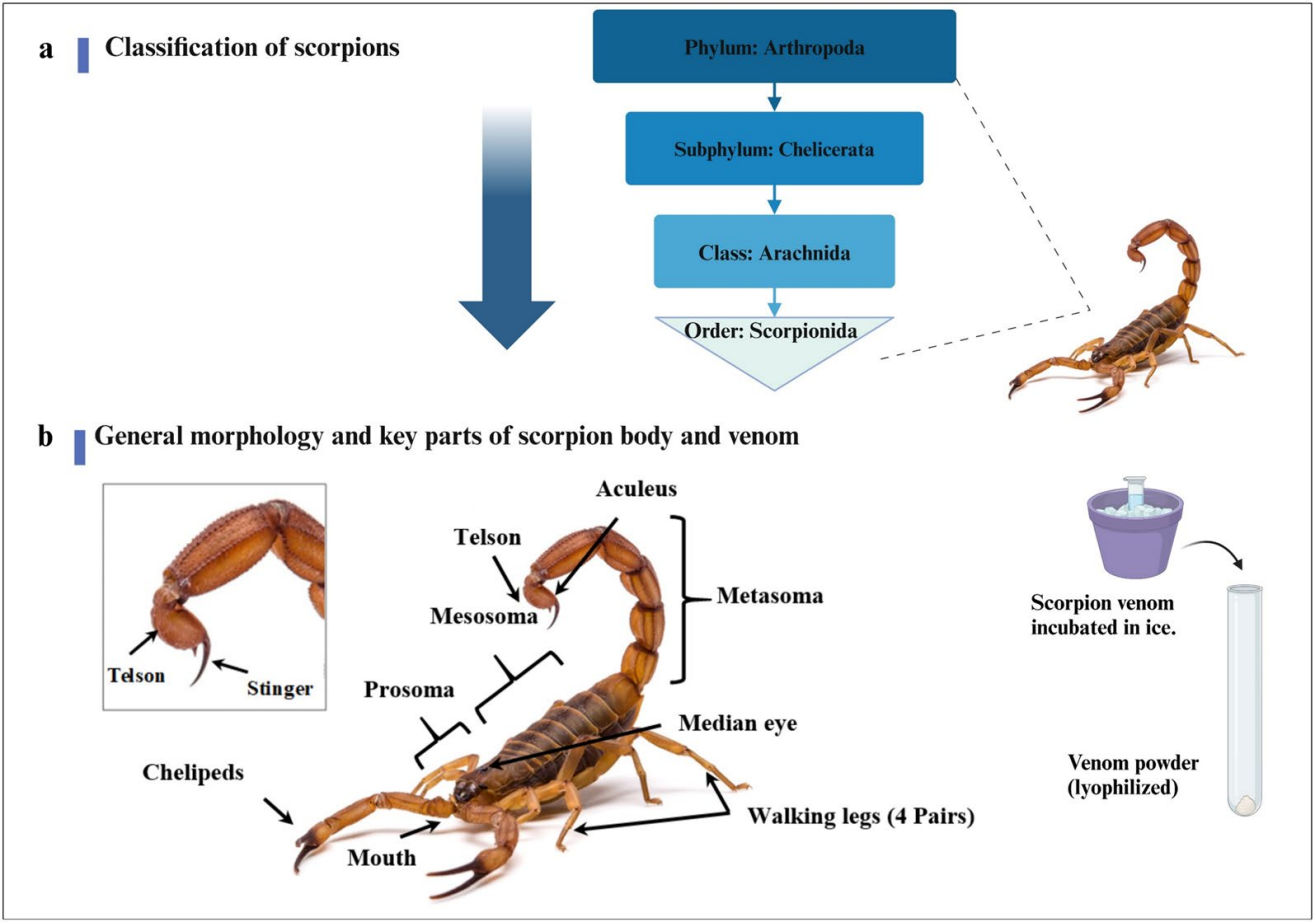
**a** Classification of scorpions, **b** General morphology and key parts of scorpion body and venom

Scorpions are the most dangerous predators among the Arachnids class (Ruiming et al. 2010) as shown in Fig. 1a. Figure 1b shows that scorpions are easily recognized by their characteristic elongated body, which is divided into an anterior region (prosoma), middle region (mesosoma), and posterior region (metasoma). Scorpions possess a specialized venom apparatus composed of a pair of venom glands connected by ducts to the aculeus at the tip of the telson on their posterior end, which produce venom for both offense and defense (Simone et al. 2021). Scorpions are terrestrial arthropods, that live mainly in deserts and can be adapted to a wide range of environmental terrains including caves, savannas, rain forests, and subtropical as well as tropical forests (Ruiming et al. 2010). They are classified phylogenetically into about 22 distinct families including over 2500 different species and subspecies. At least 25 species (fewer than 1%) are considered poisonous to humans (Ahmadi et al. 2020). The Buthidae family members contain many fatal scorpions (more than 70 genera and 770 species) represented by the following genera *Androctonus*, *Buthus*, *Leiurus*, *Mesobuthus, and Parabuthus* (Soleglad et al. 2003).

## Pathological effects of scorpion venom

**Fig. 2:**
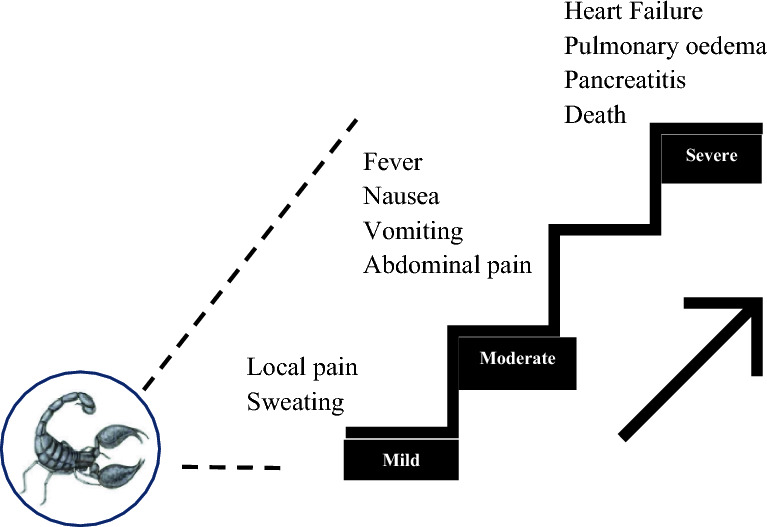
Clinical signs and symptoms of mild, oderate, and severe scorpion stings

According to global public health data, about one million scorpion envenoming are recorded annually worldwide, resulting in approximately 3000 deaths (Ward et al. 2018). The pathology of scorpion stings ranges from mild local inflammatory reactions (Cupo 2015) to moderate and severe envenoming causing heart failure, pulmonary edema, and pancreatitis, which may provoke lethal systemic responses, Fig. 2 (Pucca et al. 2016).

Despite the mortality associated with their stings, scorpions have been the target of much research interest due to their observed medicinal benefits and a wide range of pharmaceutical activities. Both crude SV and its derived proteins and peptides were used in applications ranging from cosmeceuticals and diagnostics to treatment of various ailments of the cardiovascular system, convulsions, cancer (Ahmadi et al. 2020; Srairi-Abid et al. 2019). Because of the lethality of scorpion toxins, the challenges associated with collecting the toxins, and the small amount of venom obtained from scorpions, marketing SV products for large-scale applications has been limited. Nevertheless, several toxin-based drugs have been approved and marketed over the last decade (Bordon et al. 2020). Of special importance is chlorotoxin (CTX) which showed remarkable target-specific activities in cancer patients (Wang et al. 2019).

## Classification and biochemical profile of scorpion toxins

**Fig. 3:**
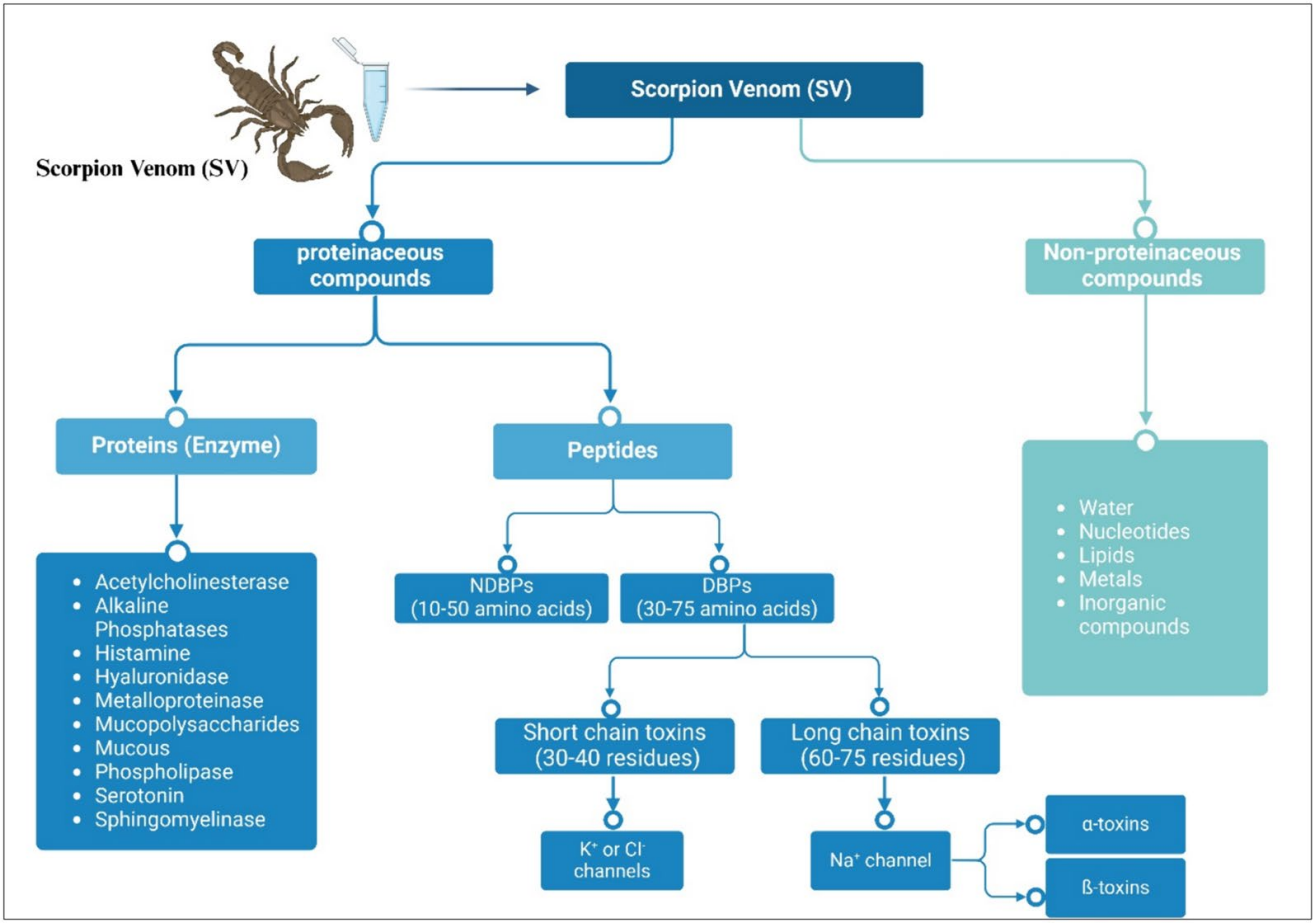
Classification of SV according to structure and effect

**Fig. 4:**
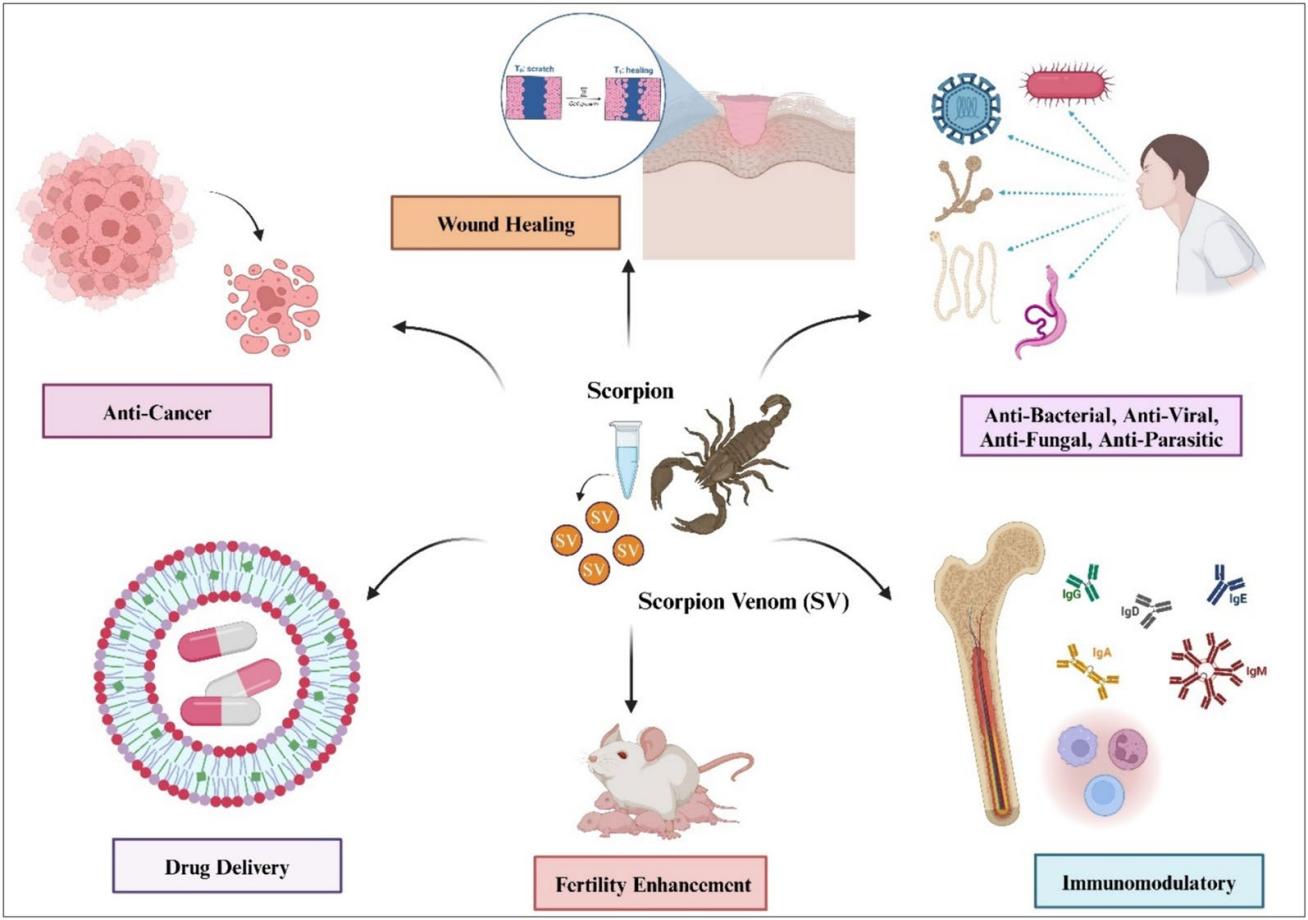
Biological properties of scorpion venom

**Fig. 5:**
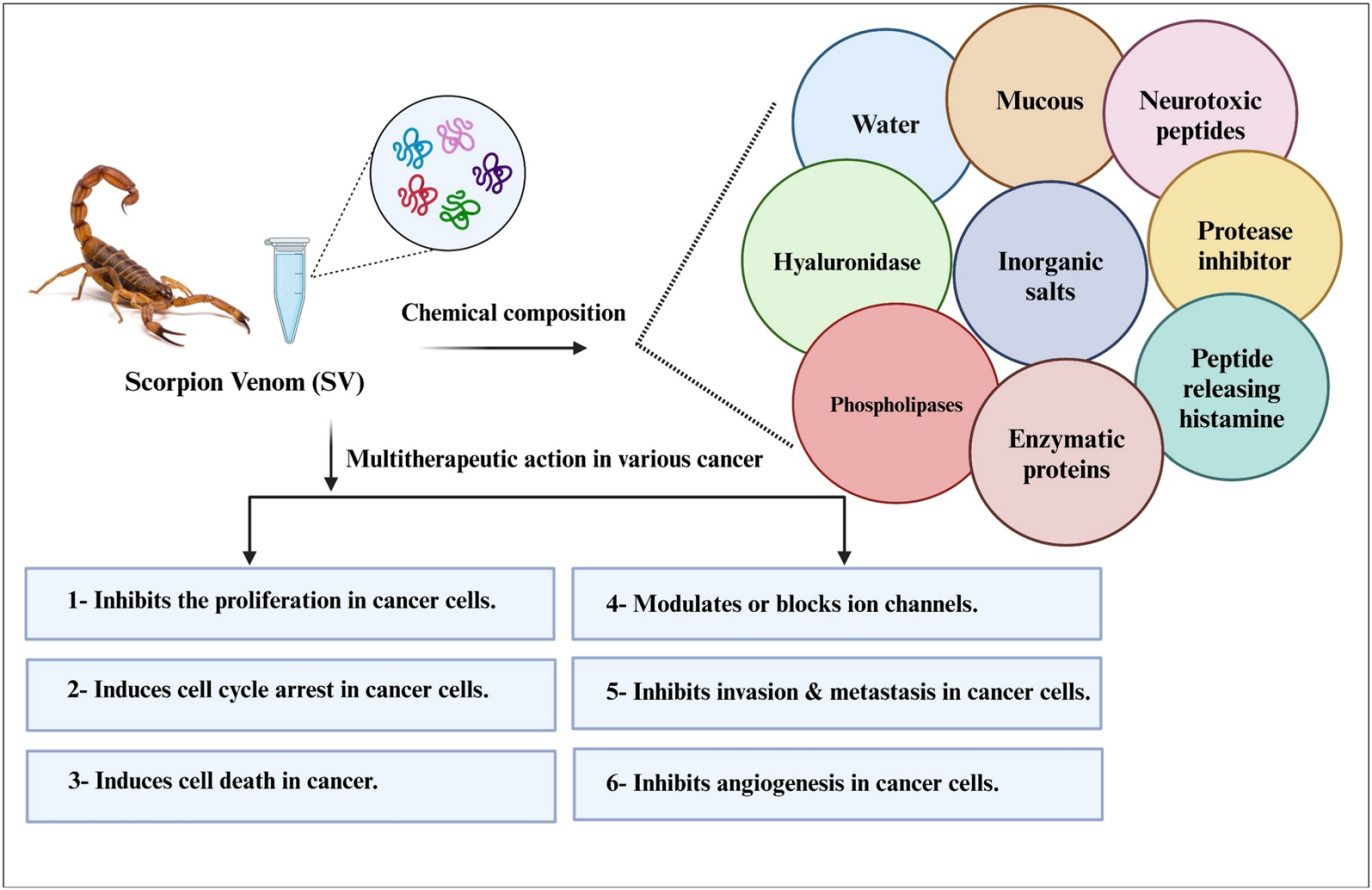
Chemical profiles of SV representing the multi-therapeutical actions of SVs and their peptides in various cancers

Scorpions employ their paralytic and lethal venom for defense and prey capture (Possani et al. 2000), although it has also been reported to be used during mating (Inceoglu et al. 2006). Scorpion toxins are classified based on the peptide length, receptor, or ion channel molecules to which the toxin specifically binds (Ca^+2^, Na^+^, Cl^−1^, and K^+^). They can be also classified based on induced receptor response (activation/blocking) and the toxin’s three-dimensional structure (DeBin et al. 1993; Dueñas-Cuellar et al. 2020; Mendes et al. 2023), Fig. 3. SV is composed of more than 500 different components including peptides that present promising sources for new pharmaceuticals, as shown in Figs. (2–6) and (2–7) (Uzair et al. 2018). Neurotoxic peptides in SV are responsible for the main pathological manifestations of envenoming. These include hyaluronidases, phospholipases, serotonin, histamine, sphingomyelinases (Cordeiro et al. 2015), acetylcholinesterase, alkaline phosphatases, proteolytic enzymes, enzyme inhibitors, mucopolysaccharides, low-molecular-weight peptides (3–10 kDa), mucoproteins, and oligopeptides. SV harbors potent neurotoxic peptides that specifically target ion channels in the nervous system that are crucial for nerve signaling, leading to paralysis (Mendes et al. 2023). Neurotoxins binding to ion channels on cell membranes cause various effects by blocking nerve impulses and modulating cell function leading to excessive stimulation or cell death (Mendes et al. 2023).

## SV in traditional medicine

The long-established tradition of using scorpion bodies and their venoms to treat ailments was reported in diverse parts of China, India, and Africa and was especially popular in Spanish folk medicine (Goudet et al. 2002). In traditional Chinese medicine, whole scorpions of the *Buthus martensii karsch* (*Bmk*) species have been used as a painkiller and to treat chronic inflammatory arthritis, spasms, convulsions, and spondylitis “Scorpio-analgesia” (Chen et al. 2022). The dry scorpion body contains components such as Makatoxin-3 which was shown to have significant pain-relieving effects (Chen et al. 2022). The venom of *Mesobuthus martensii* species, which is abundant in Eastern Asia was effectively used to treat chronic pain, rheumatoid arthritis, epilepsy, and apoplexy (Ahmadi et al. 2020; Monge-Fuentes et al. 2015). Interestingly, SV itself can be a source of antivenom. For instance, in Sudan, scorpion stings are treated by processed or diluted SV, or by immersing the whole scorpion body in sesame oil and applying this infusion to the site of the sting (Cloudsley-Thompson 1993). Traditional medicine practices have explored SV for a wider range of ailments, including cancer, infections, malaria, and immune disorders (Ling et al. 2019). Studies from traditional Chinese medicine showed that SV contained anti-cancer ingredients. For example, peptides purified from the Chinese scorpion *Buthus martensii karsch* (*Bmk*) (Jia et al. 2024; Shao et al. 2014), which a history back to the Song Dynasty of China (960–1279 AD) and well known as having potential on epileptic (Zhou et al. 1989), analgesic (Shao et al. 2014), rheumatic and cancer. *Bmk* displayed dose-dependent effects on MCF-7 cells and anti-proliferative effects on a panel of cancer cell lines (Gao et al. 2010). *BmK* prevented human lymphoma cells (Jurkat and Raji) growth and arrested the cell cycle at the Gθ/G1 phase triggering apoptosis (Heinen et al. 2011). Many of these studies however lack appropriate risk aversion, as improper preparation or use can lead to precarious outcomes and use can be dangerous and result in significant side effects. Deviation from recommended temperature or dosage protocols was also reported to cause unexpected adverse effects (Ahmadi et al. 2020). These include local reactions in the form of pain, swelling, redness, and itching, allergic reactions, and muscle tissue damage (muscle necrosis) caused by myotoxins in the SV (Ahmadi et al. 2020). Resurgent interest in SV and SVPs as remedies for cancer however is fuelled by the increasing spread of the disease and inadequate current therapy.

## SV: a natural compound with biological properties and a range of applications in cancer research and drug discovery

### Biological properties of SV and their derived peptides

#### Anti-microbial activity

SV and their peptides have been shown to exert a multitude of therapeutic effects, as shown in Fig. 4, because of their specific binding affinity to ion channels (Mendes et al. 2023; Quintero-Hernández et al. 2013) such as chloride (Lippens et al. 1995), potassium (Giangiacomo et al. 2004), and sodium (Possani et al. 2000). A significant number of non-disulfide-bridged peptides (NDBPs) from SV have demonstrated anti-bacterial properties (Table 1). For example, BmTXKS-2, a peptide purified from *Buthus martensii* triggered an antibacterial activity against *N. gonorrhoeaes* (Shao et al. 2014). Smp-24 (2578 Da) and Smp-43 (4654.3 Da), isolated from *Scorpio maurus palmatus*, demonstrated anti-bacterial activity that was specifically effective against Gram-positive bacteria, albeit of limited activity against *C. albicans* (Harrison et al. 2016). Among 11 scorpion venom-derive non-disulfide-bridged peptides, ToAP-1, ToAP-2, and ToAP-3 purified from *T. obscuru* showed effectiveness against human pathogens *Cryptococcus neoformans* and *Candida* spp. (Guilhelmelli et al. 2016). SV-derived peptides have shown inhibitory effects on various bacteria (Symeonidou et al. 2018). SV peptides such as *A. Aeneas* and *T. stigmurus* were reported to inhibit the viral infection spread (Krawczyk et al. 2013; Thakur et al. 2012), while others displayed anti-fungal properties (Table 2).

**Table 1:** SV-derived peptides with anti-bacterial activities

Peptide/venom	Sequence	Species	Dose	(Activity) anti-bacterial effect against	Reference
Serrulin	GFGGGRGGFGGGRGGFGGGGI-GGGGFGGGYGGGKIKG	*T. serrulatus* (hemolymph)	(0.5–1) μM	Gram-positive bacteria (*Micrococcus luteus* A270)	(de Jesus Oliveira et al. 2019)
Scorpine or Panscorpine (KBX3_PANIM)	GWINEEKIQKKIDERMGNTVLGGMAKAIV HKMAKNEFQCMANMDMLGNCEKHCQT SGEKGYCHGTKCKCGTPLSY	*Pandinus imperator *(Scorpionidae)	1 μM	Gram-positive and -negative bacteria	(Luna-Ramirez et al. 2017)
Stigmurin 1795.22 Da (0)	FFSLIPSLVKGLISAFK	*T. stigmurus*	1 µg/mL	Gram-positive bacteria including *S. aureus* and Methicillin-resistant *S. aureus* (MRSA)	(de Melo et al. 2015)
Marcin-18 2135.63 Da	FFGHLFKLATKIIPSLFR	*M. martensii*	1.5 μM	*Staphylococcus aureus*	(Zerouti et al. 2021)
Megicin-18 2068.04 Da	FFGALFKLATKIIPSLFR	*M. gibbosus*	1.5 μM	*Staphylococcus aureus*	
Meucin-18 2107.13 Da (0)	FFGHLFKLATKIIPSLFQ	*M. eupeus*	1.5 μM	*Staphylococcus aureus*	
Um3	GFWGKLWEGVKSAI	*U. manicatus*	2 µM	Gram-positive and -negative bacteria	(Luna-Ramirez et al. 2017)
Um5	IFKAIWSGIKSLF	*U. manicatus*	2 µM	Gram-positive and -negative bacteria	
StigA16 1949 Da	FFKLIPKLVKGLISAFK	*T. stigmurus*	2.34 µM	Gram-positive bacteria (*S. aureus*)	(Luna-Ramirez et al. 2017)
StigA6 1908 Da	FFSLIPKLVKGLISAFK	*T. stigmurus*	2.34 µM	Gram-positive bacteria (*S. aureus*)	
Meucin-49 5574.93 Da	MNKKILLVIFIVTMLIVDEVNSFKFGSFIKRMWRSKLAKKLRAKGKELLRDYANRVLSPEEEAAAPAPVPAKRRR	*M. eupeus*	3.07 μM	*Staphylococcus aureus* J706	(Zerouti et al. 2021)
Um2	ISQSDAILSAIWSGIKSLF	*U. manicatus*	4 µM	Gram-positive and -negative bacteria	(Luna-Ramirez et al. 2017)
UyCT1	GFWGKLWEGVKNAI	*U. yaschenkoi*	4 µM	Gram-positive and -negative bacteria	
BmTXKS2 (BmKb1) (BmKb2)	MEIKYLLTVFLVLLIVSDHCQAFLFSLIPSAISGLISAFK MEIKYLLTVFLVLLIVSDHCQAFLSSLIPSAISGLISAFK	*Buthus martensii*	4.3 µM	Grame-negative bacteria	(Shao et al. 2014)
LaIT2 6628.2 Da (3)	AKKPFVQRVKNAASKAYNKLKGLAMQSQYGCPIISNMCEDHCRRKKMEGQCDLLDCVCS	*L. australasiae*	7 µM	*E. coli*	(Luna-Ramirez et al. 2017)
Um4	FFSALLSGIKSLF	*U. manicatus*	8 µM	Gram-positive and -negative	(Luna-Ramirez et al. 2017)
UyCT3	ILSAIWSGIKSLF	*U. yaschenkoi*	8 µM	Gram-positive and -negative bacteria	
Smp-43 4654.3 Da (0)	GVWDWIKKTAGKIWNSEPVKALKSQALNAAKNFVAEKIGATPS	*Scorpio maurus palmatus*	5, 10 µg/mL	Highest activity against Gram-positive bacteria, limited activity against *C. albicans*	(Harrison et al. 2016)
Ctriporin	MDSKYLFVFLIFNVIVIDLCQGFLWGLIPGAVTSLIAISK	*Chaerilus tricostatus*	10 μg/mL	Gram-positive bacteria	(Almaaytah et al. 2014)
UyCT5	IWSAIWSGIKGLL	*U. yaschenkoi*	15 µM	Gram-positive and -negative bacteria	(Luna-Ramirez et al. 2017)
Uy17	ILSAIWSGIKGLL-NH_2_	*U. yaschenkoi*	23.2 µM	*Staphylococcus aureus*	
Uy234	FPFLLSLIPSAISAIKRL-NH_2_	*U. yaschenkoi*	29.6 ± 25 µM	*Staphylococcus aureus*	(Luna-Ramirez et al. 2017; Pedron et al. 2017)
AaeAP1 2016.18 Da (0) AaeAP2 1986.15 Da (0)	FLFSLIPSVIAGLVSAIRNa	*A. aeneas*	32 mg/L	*Staphylococcus aureus*	(Du et al. 2015)
	FLFSLIPSAIAGLVSAIRNa	*A. aeneas*			
Uy192	FLSTIWNGIKGLL-NH_2_	*U. yaschenkoi*	42.4 µM	*Staphylococcus aureus*	
ToAP2 9486 Da	FFGTLFKLGSKLIPGVMKLFSKKKER	*T. obscurus*	50; 12.5; 200; > 400; 25 µM	*Mycobacterium massiliense*	
Cm38 2149 Da (2)	ARDGYIVDEKGCKFACFIN	*C. margaritatus*	64 μM	*Klebsiella pneumonia*	(Dueñas-Cuellar et al. 2015)
Smp-24 2578 Da (0)	IWSFLIKAATKLLPSLFGGGKKDS	*Scorpio maurus palmatus*	64 μM	Highest activity against Gram-positive bacteria, limited activity against *C. albicans *	(Harrison et al. 2016)

**Table 2:** SV-derived peptides with anti-fungal activities

Peptide/venom	Sequence	Species	Dose	Anti-fungal activities against	Reference
Ts-1 (8300 Da)	KEGYLMDHEGCKLSCFIRPSGYCGRECGIKKGSSGYCAWPACYCYGLPNWVKVWDRATNKC	*T. serrulatus*	(1.5, 3 and 6 µg/well, which correspond to 2.18, 4.36, and 8.72 µM, respectively)	Inhibited the fungal growth of *A. nidulans*	(Santussi et al. 2017)
StigA-16 (1949 Da)	FFKLIPKLVKGLISAFK	*T. stigmurus*	1.17–9.38 µM	Inhibited fungal growth of *C. albicans*, *C. glabrata* and *C. krusei*	(Parente et al. 2018)
ToAcP	EEDDLLGFSEEDLKAIKEHRAKNA-NH_2_	*T. obscurus*	12.5 µM	Reduced the viability of *Candida sp.* as well as *Cryptococcus neoforman*	(Guilhelmelli et al. 2016)
ToAP-2	FFGTLFKLGSKLIPGVMKLFSKKKER	*T. obscurus*	12.5 µM	Reduced the viability of *Candida sp.* as well as *Cryptococcus neoforman*	(Guilhelmelli et al. 2016)
Serrulin (3564 Da)	GFGGGRGGFGGGRGGFGGGGI-GGGGFGGGYGGGKIKG	*T. serrulatus (hemolymph)*	12–24 µg/mL (3–6 µM) for *Aspergillus niger*, and 6–12 µg/mL (1.5–3 µM) for *Candida albicans*	Reduced the growth of *A. niger* and *C. albicans*	(de Jesus Oliveira et al. 2019)
ToAP-3	FIGMIPGLIGGLISAIK-NH_2_	*T. obscurus*	25 µM for *Candida sp,* and 100 µM for *Cryptococcus neoforman*	Reduced the viability of *Candida sp.* as well as *Cryptococcus neoforman*	(Guilhelmelli et al. 2016)
AaeAP-1 (2016.18 Da)	FLFSLIPSVIAGLVSAIRNa	*A. aeneas*	32 mg/L	Decreased four-fold (16 > 4 mg/L) and eight-fold (32 > 4 mg/L), respectively against* C. albicans*	(Du et al. 2015)
AaeAP-2 (1986.15 Da)	FLFSLIPSAIAGLVSAIRNa	*A. aeneas*		Decreased four-fold (16 > 4 mg/L) and eight-fold (32 > 4 mg/L), respectively against* C. albicans*	(Du et al. 2015)
StigA-6 (1908 Da)	FFSLIPKLVKGLISAFK	*T. stigmurus*	1.17–37.5 µM	Inhibited fungal growth of *C. albicans*, *C. glabrata* and *C. krusei*	(Parente et al. 2018)
ToAP-1	FIGMIPGLIGGLISAFK-NH_2_	*T. obscurus*	50 µM for *Candida sp,* and 25 µM for *Cryptococcus neoforman*	Reduced the viability of *Candida sp.* as well as *Cryptococcus neoforman*	(Guilhelmelli et al. 2016)
ToAP-4	FFSLIPSLIGGLVSAIK-NH_2_	*T. obscurus*	50 µM for *Candida sp,* and 25 µM for *Cryptococcus neoforman*	Reduced the viability of *Candida sp.* as well as *Cryptococcus neoforman*	(Guilhelmelli et al. 2016)
Hypotensin/or TistH (2700 Da)	ADMDFTGIAESIIKKIKETNAKPPA	*T. stigmurus*	MIC 128 mg/mL	Showed growth inhibition against *C. albicans*, *C. tropicalis* and *Aspergillus flflavus*	(Machado et al. 2016)
Stigmurin (1795.22 Da)	FFSLIPSLVKGLISAFK	*T. stigmurus*	> 150 µM	Inhibited fungal growth of *C. albicans*, *C. glabrata* and *C. krusei*	(de Melo et al. 2015)

#### Immunosuppressive activity of SV-derived peptides

Immune cells, similar to cancer cells, express voltage-gated potassium channels (KV) designed to control various physiological functions and immunological responses (Petricevich 2004). In immune cells, charged ions inserted within the hydrophobic membrane pores through ion channels help in the regulation of negative membrane potential (Vm), which indirectly controls Ca^+2^ ion influx and immune cell signaling/activation (Feske et al. 2015). One specific example is the KV1.3 potassium channel in T Helper 17 (Th17) cells, known to be a critical regulator of autoimmune disorders by controlling calcium ion influx (Zhao et al. 2015). Neurotoxic substances in SV were reported to directly and indirectly modulate voltage-gated Na^+^, K^+^, and Ca^+2^ ion channels (Quintero-Hernández et al. 2013). SV and its peptides interact with potassium channels and preferentially trigger KV1.3 to modulate channel expression and plasma membrane activity (Zhao et al. 2015). Studies on SV-derived peptides with immunosuppression activity that target ion channels and their clinical importance (Al-Asmari et al. 2018b; Jacoby et al. 2010; Kampo et al. 2019; Song et al. 2012) are summarized in Table 3.

**Table 3:** SV-derived peptides with immunosuppressive activities

Peptide/venom	Sequence	Species	Dose	Immunosuppressive activities	Reference
Osk-1 (4.22 KDa)	GVIINVKCKISRQCLEPCKKAGMRFGKCMNGKCHCTPK	*Orthochirus scrobiculosus*	Kv1.1: 0.6 nM Kv1.2: 5.4 nM K_Ca_3.1: 225 nM	Inhibits KV1.3 more than KV1.1, and KV1.2 channels	(Gubič et al. 2021)
Margatoxin (MgTx)	TIINVKCTSPKQCLPPCKAQFGQSAGAKCMNGKCKCYPH	*Centruroides margartatus*	1 nM	Blocks KV1.3 channels	(Feske et al. 2015)
St-20 (α-KTx)	TKCSGSPECVKFCRNGKCMNRSCKCYLCS	*Scorpions tibetanushas*	(0, 1, 10, 100 nM)	Decreases CD69 expression and the release interleukin-2 IL-2, tumor necrosis factor (TNF-α), and IFN-γ in activated human T cells	(Padmanabhan and Prince 2006)
ImKTx-88	QIYTSKECNGSSECYSHCEGITGKRSGKCINKKCVCYR	*Isometrus masculatus* (Im)	100 nM, 1, and 10 μM	Specifically inhibits KV1.3 channels	(Huang et al. 2017)
*Bmk* -AGAP	VRDGYIADDKNCGRNAYCDDECEKNGAESGYCQWAGVYGNACWCYKLPDKLPDKVPIRVPGKCNGG	*Buthus martensi Karsch *(Chinese scorpion)	0, 5, 10, 15, 20, 25, 30, 35, 40, 45, 50, 55, and 60 µM	Specially binds to Nav 1.5 channels	(Kampo et al. 2019)
*Bmk* fraction (SVCIII) (70–80 KDa)	CGPCFTTDANMARKCRECCGGIGKCFGPQCLCNRI		0, 1, 5, 10, 20, 30, 40 and 50 µg/ml	Inhibits NF-κB activation through inhibition of IκBα phosphorylation, degradation, and p65 nuclear translocation	(Song et al. 2012)
Ts-6	WCSTCLDLACGASRECYDPCFKAFGRAHGKCMNNKCRCYT	*T. serrulatus*	50, 250, 1250 nM	Both inhibit the function and proliferation of several T-cell subgroups in vitro by blocking KV2 channels	(Pucca et al. 2016)
Ts-15	GKFGKCKPNICAKTCQTEKGKGMGYCNKTECVCSEW				

## Anti-cancer activity of SV peptides

Cancer cells have been shown to use ion channels (Mikaelian et al. 2020) in their progression and metastasis, as shown in Fig. 5, via the modification in the cell volume and morphology (Capatina et al. 2022). For instance, in glioma, Cl^−^ and K^+^ ions cause electrochemical efflux mediated by intracellular Ca^+2^ ion increase resulting in tumor shrinkage (McFerrin et al. 2006; Sontheimer 2008). In breast cancer, the Ca^+2^ channels᾿s deactivation was reported to prevent tumor growth and proliferation (Aydar et al. 2009). Similarly, Cl^−^ channels were dysregulated in human colorectal cancer (Bustin et al. 2001), and modified in glioma to regulate the migration and invasion of cancer cells (Ullrich et al. 1996). Voltage-gated potassium channels-Kv and/or calcium-activated potassium channels-KCa and their subtypes were shown to be over-expressed or dysregulated in multiple cancers including glioblastoma (Griffin et al. 2020), breast (Northcott et al. 2012), colon, and prostate cancer (Comes et al. 2013) and lymphoma (Comes et al. 2015). Other studies showed that voltage-gated sodium channels (VGSCs) Nav1.5, Nav1.6, and Nav1.7 were also over-expressed in many cancers like breast cancer, colon cancer, cervical cancer, prostate cancer, and lung cancer. This overexpression displays as an important player in migration and invasion and has a distinct role in nerve signal transmission (Brisson et al. 2013), and presents a selective target for potent compounds with better-targeted drug delivery (Bordon et al. 2020; Uzair et al. 2018). Among these compounds, chlorotoxin (CTX) is a small venomous peptide (36 a.a.) that was first isolated in 1993 from *Leiurus quinquestriatus* yellow scorpion (*L. quinquestriatus*). CTX is best known for its selective binding affinity to chloride channels on the surface of glioma cells (DeBin et al. 1993). It disrupts the cancer cell's ability to invade surrounding tissue by blocking the influx of chloride ions, which are essential for changes in cell shape and volume required for cell invasion (Deshane et al. 2003; McFerrin et al. 2006; Soroceanu et al. 1999). Notably, this effect appears to be specific to glioma cells and has not been observed in healthy cells (DeBin et al. 1993). Binding CTX to Cy5.5 fluorescence dye (CTX-Cy5.5 conjugated tumor-targeting peptide) enabled visualization of the tumor site (Veiseh et al. 2007) and facilitated precise surgery and targeted therapy without damaging healthy cells (Boltman et al. 2023). CTX conjugated with nanoparticles was shown to be effective in depositing drugs at specific tumor sites (Fu, Y. et al. 2012). CTX was conjugated with Iron oxide nanoparticles and targeting ligands, all connected via a polyethylene glycol (PEG) linker. This design facilitated preferential accumulation and enhanced cytotoxicity in glioblastoma and neuroblastoma cells (Boltman et al. 2023). CTX-conjugated with magnetic nano chains could target non-small-cell lung cancer A549 cells, leading to the release of free radicals and the production of reactive oxygen products (ROS). As a result, a significant increase in cancer cytotoxicity and a halt in tumor progression was observed when compared to CTX treatment alone (Zhao et al. 2011). SVPs products including CTX toxins (Wang et al. 2019) further their anticancer effect by altering molecular targets in cancer cells (Desales-Salazar et al. 2020), penetrating the cell membrane of the tumor cells (Veiseh et al. 2007), and attaching to other drug carriers for drug delivery (Chung et al. 2023), such as nanoparticles (Chung et al. 2023), liposomes (Li et al. 2021), and oligonucleotides (Chen et al. 2020).

### Tumors of the nervous system

*Odontobuthus doriae*’s venom was reported to induce swelling and rupture of the neuroblastoma cell membrane and increase the release of cytosolic materials (Zargan et al. 2011b). SV-treated neuroblastoma cells exhibited an increase in lactate dehydrogenase (LDH), compromised cell viability, and up-regulation of caspase 3 leading to DNA damage, inhibited DNA synthesis, apoptosis, and necrosis. *Bmk* -peptide (*Bmk*-CTx) from *Buthus martensii Karsch*-*Bmk* upregulated tumor suppressor p53 protein in human glioblastoma U87-MG cells in vitro, as shown in Table 4 (Wu, S. et al. 2018), and in vivo study by xenografting U251-MG tumor in SCID mouse models Table 5. Notably, p53 protein was increased after *Bmk* -peptide treatment.

**Table 4:** In vitro anti-cancer activities of SV and SVPs and their mechanisms of action on human cancer cells

Peptide/venom	Sequence	Dosage	Species origin	Activity	Cancer type	Cell line	Target	Reference
Crude venom	–	Dose dependant manner	*A. crassicauda*	Proliferation blocking	Human neuroblastoma	SH-SY5Y	Induces apoptosis through increasing nitric oxide production, caspase-3 activity, and depolarizing mitochondrial membrane and arrests S phase	(Zargan et al. 2011a, 2011b; Zargan et al. 2011a, b, c)
					Human breast cancer	MCF-7		
AcrAP-1 and AcrAP-2 (non-disulfide-bridged peptides) (NDBP), and their high cationic analogs (HC-AcrAP)	FLF KLIP KAI KGLI KAFK FLF KLIP KAI KGLL KAFK	IC_50_ (0.001-100 µM)	*A.* *crassicauda*	HC-AcrAP analogs proliferation blocking (IC50 2–3.6 µM)	Human lung adenocarcinoma. Human breast carcinoma. Human breast tumorigenic. Human prostate carcinoma	H460 MB435s MCF-7 PC-3	(Probably) inducing cell lysis	(Possani et al. 2000)
Bengalin 72 kDa	GPLTILHINDVHAAFEQFNT	IC_50_ more than 3 (3.5–4) µg/mL	*Heterometrus bengalensis koch*	Proliferation blocking	Human leukemia	U937 and K562	Increases expression of Bax/Bcl-2 ratio, caspase 3 and 9, reduces mitochondrial membrane potential, heat shock proteins (HSP) 70 and 90	(Gupta et al. 2010; Heather et al. 2016)
*Bmk* (70-80 kDa)	CGPCFTTDANMARKCRECCGGIGKCFGPQCLCNRI	5. 10 µM	*B martensii Karsch*	Proliferation blocking	Human leukemia	(THP-1) acute monocytic leukemia cell line	Inhibit the invasion ability of U87 glioma cell	(Song et al. 2012)
AaCTX	MCIPCFTTNPNMAAKCNACCGSRRGS–CRGPQCIC–-	5. 200 µM	*Androctonus australis*	Inhibition the migration and invasion	Human glioma cells	U87	Inhibit U87 glioma cell migration	(Rjeibi et al. 2011)
*Bmk* -AGAP 71.42KDa (66 amino acids)	VRDGYIADDKNCGRNAYCDDECEKNGAESGYCQWAGVYGNACWCYKLPDKLPDKVPIRVPGKCNGG	(IC_50_ = 40 µM for MCF-7 and 50 µM for MDA-MB-231 cells)	*B martensii Karsch*	Proliferation blocking, stemness, sphere formation, colony formation epithelial-mesenchymal transition, migration, and invasion	Human breast cancer	MCF-7 and MDA-MB-231	Down-regulation of Oct4, SOX2, Nanog, N-cadherin, Snail, and PTX3 and up-regulation of E-cadherin at both gene and protein levels Interference of Na^+^ toxin with P-AKT, NF-kB, Bcl-2, and MAPK signaling pathway	(Kampo et al. 2019)
Crude venom		IC_50_ (20-100 µg/mL)	*A bicolor* *A crassicauda*	Prevents cell motility and colony mitosis	Human breast cancer	MDA-MB-231	Two mechanisms that involved in migration and invasion of cancer cells are proposed a) a decrease in MMPs expression, and b) a reduction in FAK autophosphorylation levels	(Al-Asmari et al. 2016a, b)
					Human colorectal cancer	HCT-8 and HCT116		(Al-Asmari et al. 2016a, b)
Gonearrestide (P13) (18 amino acids, 2192 Da)	W C Y K L P D R V S I K E K G R C N	IC_50_ (2-200 µM)	*A mauritanicus*	Proliferation blocking in dose-dependent	Human colon cancer	HCT116	Arrests cancer cell cycle in the G1 phase via modulating cell cycle checkpoint proteins (down-regulate CDK4, and upregulate cyclin D3, p21, p27)	(Li et al. 2018)
*Bmk* (50–60) amino acids 65 kDa	VRDAAYIAKPENCVYECGITQDCNKLCTENGAESGYCQW	IC_50_ (10-200 µg/mL)	*B martensii Karsch*	Proliferation blocking	Human Prostate cancer	DU145	Upregulation of Bax apoptotic gene expression, and down-regulated of Bcl-2 anti-apoptotic gene expression	(Zhang et al. 2009)
							Inhibiting proliferation by G1/S arresting cancer cells cycle	(Kampo et al. 2019)
*Bmk* -CT	CGPCFTTDANMARKCRECCGGIGKCFGPQCLCNRI			Migration and invasion blocking	Human glioma	SGH-44	Blocking of Cl^−1^ ion channel, hence, selectively target glioma	(Hmed et al. 2013; Jian 2014; Pedersen and Stock 2013)
Iberiotoxin (IbTX) (34 amino acids, 36.07 kDa)	GDCLPHLKRCKADNDCCGKKCKRRGTNAEKRCR		*Buthus tumulus*	Prevents growth and proliferation	Human cervical cancer	HeLa	Blocks calcium-activated potassium channels KCNMA1 (KCa1.1, BK) Found to be expressed in cervical cancer-hormone dependent block large conductance Ca^+2^ activated K^+^ (BK) channel	(Han et al. 2007; Ramírez et al. 2018)
					Human ovarian cancer	A2780		
					Human breast cancer	MCF-7		
Margatoxin (MgTX, 39 amino acids, 41.92 kDa)	TIINVKCTSPKQCLPPCKAQFGQSAGAKCMNGKCKCYPH	Dose dependant manner	*C. margartatus*	Proliferation blocking	Human lung adenocarcinoma	A549	Blocks Kv1.3 and increases expression level of p21Waf1/Cip1 and decreases the expression level of Cdk4 and cyclin D3	(Jang et al. 2011)
Chlorotoxin (CTX/ or ClTx) (36 amino acids, MW4.02 KDa)	MCMPCFTTDHQMARKCDDCCGGKGRGKCYGPQCLCR	IC_50_ more than 20 (20–100) µg/mL	*L quinquestriatus*	Stops migration and invasion	Human glioma	D54-MG and CCF-STTG-1	Specially binds to MMP-2 and modulates active MMP-2 expression and enhance Cl-channel receptors uptake (CIC-3)	(Deshane et al. 2003; McFerrin et al. 2006)
				prevents cell motility, and colony mitosis	Human breast cancer	MDA-MB-231		
crude venom	–		*L quinquestriatus*		Human colorectal cancer	HCT-8 and HCT116	Two mechanisms proposed are a) a decrease in the expression of MMPs, and b) a reduction in the phosphorylation levels of FAK, which is involved in cell migration and invasion	(Al-Asmari et al. 2016a, b)
crude venom	–	IC_50_ (20–100) µg/mL	*O doriae*	Prevents proliferation and DNA synthesis	Human breast cancer	MCF-7	Depolarizes mitochondria, activates Caspase-3, and depletes antioxidant activities	(Zargan et al. 2011a, b, c)
					Human neuroblastoma	SH-SY5Y		Zargan et al. 2011a, b, c)
crude venom	–	IC_50_ (100–1000) µg/mL	*R junceus*	Proliferation blocking	Different human cancer cell lines	HeLa, SiHa, Hep-2, NCI-H292, A549, MDA-MB-231, MDA-MB-468, HT-29	Increased expression of P53, Bax, Caspase 3, 8, & 9 and reduced Bcl-2 in HeLa (apoptosis > necrosis) whereas reduced expression p53, did not affect Bax but reduced Bcl-2 in A549 (necrosis > apoptosis) reflecting concentration used > IC_50_ or < IC_50_	(Mikaelian et al. 2020)
Crude venom		IC_50_ (500-1000 µg/mL)	*A bicolor* *A crassicauda*	Proliferation blocking	Human breast cancer. Human colorectal cancer	MDA-MB-231 HCT	Induces apoptosis more than necrotic death, and arrests cells at G0/G1 phase upregulates p53, downregulates Bcl-xL	(Al-Asmari et al. 2016a, b; Al-Asmari et al. 2016a, b; Al-Asmari et al. 2018a, 2018b)
Neopladine-1 (29.918 kDa) and Neopladine-2 (30.388 kDa)	*–*	Dose dependant manner	*T discrepans*	Both peptides induce apoptosis	Human breast carcinoma	SKBR3	Bind to SKBR3 cell surface and trigger FasL and BcL-2 expression	(D’Suze et al. 2010)
TsAp-1 and TsAp-2 (17 amino acids) high cationic TsAP-1 and TsAP-2	ATGCAAATAAAACATCTCATTACTCTCTTCTTTCTCGTCTTGATCGTTGC	Dose dependant manner	*T serrulatus*	Highly proliferation inhibitors	Human squamous carcinoma lung adenocarcinoma. Human prostate adenocarcinoma Human breast carcinoma. Human glioblastoma	H157 H838 PC-3 MCF-7 U251-MG		(Guo et al. 2013)

**Table 5:** In vivo anti-cancer effect of SV and SVPs and their mechanisms of action

Peptide/venom	Sequence	Dosage	Duration	Species origin	Activity	Mechanism	Reference
Crude venom	–	0.22 mg/kg/day i.p. (20% LD dose)	13 days	*A amoreuxi*	Decreasing Ehrlich ascites carcinoma and solid Ehrlich tumor size, but mice-bearing intraperitoneal tumor survival increasing	Down-regulates Ki-67 and VEGF expression and up-regulates Caspase-3 expression	(Salem et al. 2016)
*Bmk* -AGAP 71.42KDa with 66 amino acids	VRDGYIADDKNCGRNAYCDDECEKNGAESGYCQWAGVYGNACWCYKLPDKLPDKVPIRVPGKCNGG	(0.5 and 1 mg/kg) injected i.p	20 days at 48 h. intervals	*B martensii*	Reduced tumor growth of MDA-MB-231 cells in mice	Reduces gene and protein expression in ex vivo tumors of Oct4, SOX2, Nanog, N-cadherin, Snail, and PTX3, and increases the expression of E-cadherin	(Kampo et al. 2019)
Crude venom	–	17.5, 35, 52.5 µg	twice a week for 16 weeks	*L quinquestriatus*	Decreased skin carcinogenesis incidence in mice	Downregulates expression of Ki-67, NF-kB, Cox-2, Bcl-2, VEGF, and pro-inflammatory cytokines (TNF-α and IL-6)	(Almaaytah et al., 2016)
TAM-601 (synthetic ClTx) TAM-601	MCMPCFTTDHQMARKCDDCCGGKGRGKCYGPQCLCR	2. 10, or 100 mg/kg (i.v.)	3x/week for 2 weeks	*L quinquestriatus*	Blocks angiogenesis in chick. Chorioallantoic membrane growing human tumors. Reduces micro-vessel count in mice using Matrigel plug assay	Inhibits VEGF, PDF, and TNF-α action on vascularization and blocks MMP-2 activity	(Jacoby et al. 2010)
Gonearrestide 18 amino acids (P13) (2192 Da)	W C Y K L P D R V S I K E K G R C N	50 and 100 µM peritumoral injection	2 weeks	*A mauritanicus*	prevents tumor growth in HCT116 (colon cancer cell line) in dose-dependent	Cell cycle checkpoint proteins modulation (down-regulates CDK4, and up-regulates cyclin D3, p21, p27)	(Li, B. et al., 2018)

Glioblastoma or astrocytoma-IV is the most uncontrolled type of primary brain cancer (Minniti et al. 2021). Current therapies entail surgical resection, radiotherapy, and chemotherapy; however, the mortality rate remains high. The post-diagnostic survival of less than 15 months is due to neoplastic cells’ migration to surrounding brain tissues (Tewarie et al. 2021), and chemotherapeutic resistance to drugs such as temozolomide (TMZ) (Jiapaer et al. 2018). CTX, purified from *Leiurus quinquestriatus* was found to specifically bind to glioma cells (Jacoby et al. 2010) sparing healthy cells (Lyons et al. 2002). It acted by preventing Cl^−1^ ions inflow across glioma cell membranes by blocking small-conductance Cl^−1^ channels (Soroceanu et al. 1998, 1999).

CITx was reported to target human D54-MG and CCF-STTG-1 glioma cells and prevent metastasis (Deshane et al. 2003; McFerrin et al. 2006). Invasion of glioma cells requires Cl^−^ ions inflow through the cell membrane (Soroceanu et al. 1999) and MMPs activation (Sawaya et al. 1996). ClTx was found to selectively bind to glioma cell-MMP-2, but not to normal glial cells, forming a ClTx-MMP-2 complex (Deshane et al. 2003). This results in MMP-2 deactivation and reduction in the gelatinase activity of the glioma cell membrane, leading to inhibition of glioma cell invasion into the surrounding brain tissues (Soroceanu et al. 1999). In vitro studies confirmed that ClTx reduced glioma cell migration by MMP-2 deactivation and reduction in Cl^−^ ions expression, thus preventing glioma cells from shrinking and migration (Dardevet et al. 2015). ClTx can also serve as a glioma-specific marker or “tumor paint” by conjugating with fluorescent dye to delineate the tumor boundaries and facilitate their surgical removal (Veiseh et al. 2007). Similarly, TM-601, a synthetic product of ClTx, retarded the growth of U87-MG glioblastoma cells (Veiseh et al. 2007) and reduced angiogenesis in chick and mouse models through the suppression of both vascular endothelial growth factor (VEGF) and platelet-derived growth factor (PDGF) signaling pathways (Jacoby et al. 2010). Iodine-131 radio-labelled ClTx peptide (^131^I-TM601, ^131^I-ClTx) was retained in patients with recurrent glioblastoma for up to 8 days after administration (Wu, X. S. et al. 2010). Experiments using ^125^I- and (^131^I)-labelled CTX injected into SCID mice bearing xenografted gliomas showed its specific affinity to glioma, and not normal, thus serving as glioma-specific markers (Soroceanu et al. 1998).

*Bmk* peptide, another SV-derived Cl^−^ channel inhibitor, was reported to inhibit human glioma SHG-44 cell growth and proliferation at 29 times lower concentration than that needed to inhibit normal astrocytes (Fu, Y. J. et al. 2007).

In an in vivo study, *Bmk* was bound to MMP-2 and inhibited the glioma C6 cells migration and invasion in rats via MMP-2 blockage (Fu, Y. J. et al. 2011; Fu, Y. J. et al. 2007). *Bmk* -AGAP is a long-chain neurotoxin with 7142 Da and 66 a.a. that is specific to the Na^+^ channel. It was shown to suppress glioma cell growth by apoptosis induction. Iberiotoxin-IbTx peptide (37 a.a.) purified from *Mesobuthus tumulus* with about 68% sequence similarity to charybdotoxin-ChTX, was reported to selectivity bind to BK channel (Ortiz et al. 2015). IbTx inhibited cell growth of human 1321N1 astrocytoma cells (Ouadid-Ahidouch et al. 2004), and caused S phase halt and apoptosis induction in glioma cells (Griffin et al. 2020). Notably, IbTx inhibited the growth of HeLa cell and human ovarian cancer (A2780) cell lines (Mikaelian et al. 2020), and in hormone-dependent cancers like breast, prostate, ovarian, and cervix cancers, IbTx peptide inhibited cancer development and growth via inhibition of calcium-activated potassium channels (KCNMA1) (Oeggerli et al. 2012; Ramírez et al. 2018). Another peptide, AaCtx is the first CTX-like peptide isolated from *Androctonus australis* scorpion venom. Its amino acid sequence shares 70% similarity with CTX, from which it differs by 12 amino acids. Due to its very low concentration in venom (0.05%), AaCtx was chemically synthesized. Both native and synthetic AaCtx were active on invasion and migration of human glioma cells. However, their activity was found to be lower than that of CTX. The molecular model of AaCtx shows that most of amino acids differing between AaCtx and CTX are localized on the N-terminal loop and the α-helix. Based on known compounds that block Cl^−1^ channels, it is suggested that the absence of negative charged amino acids on AaCtx structure may be responsible for its weak activity on glioma cells migration and invasion. This finding serves as a starting point for structure–function relationship studies leading to design high specific anti-glioma drugs (Rjeibi et al. 2011).

### Hematologic malignancies

In various hematopoietic malignancies including leukemia, lymphoma, and multiple myeloma, SV and their peptides were shown to modulate the NF-kB signaling pathway that is accountable for cancer cell growth and proliferation, and immune cell development and function, as shown in Table 4. (Escárcega et al. 2007). Activation of the NF-kB signaling pathway by SV showed selective binding to human leukemia Jurket cell line and THP-I cells inducing apoptosis (Hayden et al. 2006). SV-derived SVCIII peptides (70–80 kDa) showed promise as an anti-cancer agent by selectively inhibiting the growth and proliferation of human leukemia (THP-1 cells) and lymphoma (Jurkat cells) cells, while having no effect on healthy peripheral blood lymphocytes. The peptide decreased cyclin D1 protein expression levels and led to G1-phase cell cycle arrest. This was associated with the deactivation of NF-kB signaling pathways through IκBα phosphorylation, degradation blocking, and p65 nuclear translocation (Song et al. 2012). In another study, Bengalin peptide purified from the Indian scorpion *Heterometrus bengalensis Koch* (Hbk) exerted cytotoxicity to K562 chronic myelogenous leukemia cells at IC_50_ values of 4.1 mg/mL. It is also human-inhibited U937 histiocytic lymphoma cells at 3.7 mg/mL without affecting healthy lymphocytes, (Gupta et al. 2010). Bengalin-mediated apoptosis was achieved by the intrinsic mitochondrial death pathway, sub-G1 phase cell cycle arrest, silencing of telomerase activity, and DNA damage (Gupta et al. 2010).

### Breast cancer

Breast cancer (BC) is one of the most pervasive cancers, accounting for nearly 1 in 8 newly diagnosed cases worldwide (Arnold et al. 2022). Hyaluronidase-BmHYA1 purified from Chinese red scorpion *B. martensi* was shown to specifically inhibit hyaluronan activity in BC cells, without observed toxic side effects (Feng et al. 2008). ClTx peptide from *L. quinquestriatus* scorpion prevented the growth of 4T1 cell line, obtained from the most metastatic and aggressive BCs (Qin et al. 2014), as shown in Table 4.R

Two novel peptides isolated from *Tityus discrepans* (Neopladine-1 and Neopladine-2) displayed effectiveness against SKBR3 BC cell line. Both peptides were shown to selectively bind to SKBR3 cell membrane, causing upregulation in BcL-2 and FasL expression in cancer cells after less than 5 h. of exposure (Ding et al. 2014). Similarly, peptides from *Odontobuthus doriae* venom induced DNA damage and apoptosis in MCF-7 BC cells (Zargan et al. 2011a, b, c). Another study reported the anti-cancer cytotoxic effect of SV-derived ICD-85 on both BC cell lines (MDA-MB-231 and MCF-7), as well as HELA HL-60 cancer cells, but not on normal human fibroblast MRC-5 cell line (Kheirandish Zarandi et al. 2019). Recombinant *Bmk*-AGAP was also shown to be effective against cancer cells by selectively binding to voltage-gated Na^+^ channels (Kampo et al. 2019).

rBmK AGAP peptide was reported to inhibit breast cancer cell stemness, epithelial-mesenchymal transition (EMT), migration, and invasion both in vitro and in vivo, as shown in Tables 4 and 5. This inhibition was mediated by down-regulating PTX3 through NF-κB and Wnt/β-catenin signaling pathways, which may be due to selective binding to one of the voltage-gated- Na^+^-channel subunits, Nav 1.5 (Kampo et al. 2019), which is over-expressed in BC and has a role in its progression (Brackenbury 2012). Selective binding of r*Bmk*-AGAP peptide to Nav 1.5 caused its upregulation and led to an increase in the pentraxin 3 (PTX3) mediator expression. This led to the deactivation of tumor necrosis factor (TNF)-α and modulation of the NF-κB pathway resulting in the preventing of BC progression (Kampo et al. 2019). Intraperitoneal administration of 0.5 or 1 mg/kg of r*Bmk*-AGAP into mice bearing xenograft of MCF-7 or MDA-MB-231 cells led to EMT and inhibition of stem-like features in both cell lines. This was achieved via down-regulation of PTX3, N-cadherin, Snail-1, Oct4, Sox2, β-catenin, pGSK3-β, Nav 1.5, and p65/NF-κB, and upregulation of E-cadherin and GSK3-β expression (Kampo et al. 2019). Compared to controls both tumor volume and weight were significantly reduced in treated mice (Kampo et al. 2019). Another anti-cancer peptide (ANTP, 6280 Da), purified from *Buthus martensi karsch* (*Bmk*) scorpion showed cytotoxicity against cancer cells in mouse tumor model with Ehrlich ascites, and in S-180 fibrosarcoma mouse model (Liu et al. 2002).

Similarly, CTX was shown to inhibit breast cancer by downregulating ERα expression levels, which suggests that CTX might disrupt a key pathway involved in breast cancer progression by interfering with the ERα signalling pathway (Wang et al. 2019). CTX directly binds to ERα and affects several globular α-helical folded structures (H1-H12) in the specific secondary of Erα, potentially leading to altered biological functions of ERα (Wang et al. 2019). Vasodilator-stimulated phosphoprotein (VASP) which regulates cell movement (Carmona et al. 2016) was reported to be the target gene of the ERα signalling pathway in the same study (Padilla-Rodriguez et al. 2018). Furthermore, CTX acts as a direct modulator of ERα function, potentially impacting its role in breast cancer progression with no effect on normal cells (Wang et al. 2019).

### Lung adenocarcinoma

Lung cancer is classified into small-cell lung cancer (SCLC) and non-small-cell lung carcinoma (NSCLC), both of which are among the most cancer-leading deaths worldwide with a low five-year survival rate (Lee et al. 2018). SV enzymes: acetylcholinesterase, alkaline phosphatase, phospholipase A2, and proteolytic enzymes with gelatinolytic and cytotoxic effects were reported to cause necrosis, hemolysis, and gangrene in lung cancerous cell lines (Almeida et al. 2002). Treatment with proteases from *Mesobuthus gibbosus* A caused remarkable toxicity in human lung adenocarcinoma A549 cell lines (Pessini et al. 2001). From *Centruroides margaritatus* species, Margatoxin (MgTX) is a highly selective K^+^ channel peptidyl inhibitor with 39 amino acids (Helms et al. 1997). MgTX showed significant inhibition of lung adenocarcinoma cell (A549 cell) growth, Table 4, and halted tumor growth in nude mice, Table 5 (Jang et al. 2011). In addition, MgTx peptide was tested to confirm its inhibitory effect and the results showed that MgTx could induce up-regulation of p21Waf1/Cip1 protein expression and downregulation of Cdk4 and cyclin D3 expression levels, both via inhibition of KV1.3 ion channels (Shahzadi et al. 2021).

### Cervical cancer

Cervical cancer is classified as the fourth most common cancer type after BC in women worldwide (Jacobs et al. 2018). In 2016, a study found that the anti-microbial peptide (AMP), TistH purified from *Tityus serrulatus* SV (TsV) showed a cytotoxic effect on SiHa primary uterine cell line Table 4, without any toxic effect on normal 3T3 embryonic mouse fibroblast cell line (Machado et al. 2016). Another study showed that treatment with crude venom of *Tityus serrulatus* scorpion (TsV) decreased HeLa cell viability in a dose-dependent manner, and increased caspase-mediated apoptosis of both SiHa and HeLa human cervical carcinoma lines, (Bernardes-Oliveira et al. 2019). An in vitro study demonstrated the anti-proliferative effect of LMWSVP, a low molecular weight peptide purified from *Bmk* crude venom, on SMMC7721 human hepatoma cells in a dose-dependent manner. LMWSVP also increased the expression of caspase-3 and decreased that of the anti-apoptotic protein Bcl-2 (Li et al. 2014).

### Melanoma (skin cancer)

Venom from the medically important scorpion *Leiurus quinquestriatus* demonstrated promising anti-cancer effects in mice. Treatment with this venom significantly inhibited the growth and number of tumors induced by DMBA and croton oil on mouse skin. Furthermore, the venom reduced the expression of pro-inflammatory cytokines and downregulated key molecular markers associated with tumor development, including Ki-67, nuclear factor kappa-B (NF-κB), cyclooxygenase-2 (COX-2), B-cell lymphoma-2 (Bcl-2), and vascular endothelial growth factor (VEGF) as revealed by immunohistochemistry analysis (Al Asmari and Khan 2016). A study demonstrated that CTX specifically binds to gliomas and a variety of peripheral tumors of neuroectodermal origin (PNET), including melanoma. bCltx is a synthetic molecule that is biotinylated at the N-terminus which was found to specifically bind to exhibit high binding affinity, targeting 50% to 100% of the cells within melanoma tissues (Lyons et al. 2002). Similarly, a study demonstrated that TM-601 molecules, a synthetic peptide also have an anti-cancer effect against SK-Mel melanoma, as shown in Table 4 (Romo Vaquero et al. 2012).

### Other cancers

Prostate cancer (PC) is the leading cancer in men worldwide (Rizzo et al. 2022). PESV is 60 amino acid polypeptide derived from *Buthus martensi Karsch* (*Bmk*) scorpion (Li et al. 2019). PESV showed cytotoxic activity on hormone-refractory, androgen-independent PC cell lines (Zhang et al. 2009)*.* In mice, PEVS also inhibited neovascularization and tumor growth in S180-sarcoma and H22-hepatocellular carcinoma, as shown in Table 5 (Zhang et al. 2009).

In gastric cancer, *Bmk* venom was shown to decrease the viability and inhibit the proliferation of NUGC-3 human gastric cancer cells significantly at a dose of 5 mg/mL, Table 4. Treatment of NUGC-3 cells with 5 mg/ml *Bmk* venom for 24 h showed a 6% increase in the sub-G1 cell cycle phase, a 9% increase in the G1 phase, and an 11% decrease in the G2/M phase, and increased cell apoptosis (Jian 2014). In oesophageal cancer, a study demonstrated the cytotoxicity of *Heterometrus liangi* scorpion venom against human oesophageal cancer cell lines KYSE-510 cell lines led to inducing cell death through apoptosis pathway, as shown in Table 4.

Pancreatic cancer is known for local invasion, early metastasis, and a strong desmoplastic reaction. An in vitro study demonstrated that CTX at a concentration of 300 nM significantly inhibited the cell migration of PANC-1 pancreatic cancer cells. The monomeric form of chlorotoxin (M-CTX-Fc) was generated by joining the CTX peptide to the amino terminus of the human IgG-Fc domain without a hinge domain. This form inhibited the migration ability of PANC-1 cells when compared with the control, suggesting that M-CTX-Fc may be effective for targeting pancreatic cancer (El-Ghlban 2014).

In colorectal cancer, a battery of *in vitro* biological functional studies and bioinformatics analyses demonstrated that Gonearrestide peptide, purified from *Androctonus mauritanicus* (Ama) exerted a potent anti-tumor effect (Liscano et al. 2020). Gonearrestide is 18 amino acids long, and 2192 Da It was also found to target a broad spectrum of cancer cells such as human colon cancer cell line HCT116, with no reported cytotoxicity on erythrocytes and surrounding epithelial cells (Li, B. et al*.*, 2018). Gonearrestide halted tumor growth and cancer cell proliferation by modulating cell cycle checkpoint proteins. It triggered cancer cell cycle arrest in the G1-phase through inhibition of cyclin-dependent kinases 4 (CDK4) and up-regulating the expression of cell cycle regulators/inhibitors—cyclin D3, p27, and p21. In vivo, xenograft mouse model treatment with Gonearrestide significantly halted the growth of xenografted human tumors in a mouse model and reduced the tumor volume in a dose-dependent manner, in Table 5 (Li, B. et al*.*, 2018).

SVPs targets in mammalian cells are related to key biological functions, many of which are related to cancer development and progress. In addition, studies now suggested that new molecules from SVs could neglectable cytotoxic effects by the isolation of each peptide by owns via unwinding the peptide bonds to facilitate their targets. Despite these successful discoveries, there is a gap between the number of peptides with interesting pharmacological properties obtained from SV. Basic research on scorpion venom faces two main limitations: the limited quantity obtained from different species and the inherent complexity of the venom itself, which makes isolating specific toxins a challenge.

## Conclusion and future directions

SVs are a rich bio-source of molecules with a wide range of biological and pharmaceutical applications. SVPs were found to be especially valuable for cancer therapy. As researchers identify and characterize an increasing number of peptides from different scorpion species, a wide range of toxic properties to cancer properties are discovered and applied to various types of malignancies, while sparing normal cells. The latter is a highly desirable character is modern approaches to cancer therapy, and to avoid the determinantal side effects of current cytotoxic therapies. Despite their high selectivity, the safety profile for new SV derived drugs requires extensive research. Only a few scorpion species have been extensively studied, but thousands remain unexplored, especially of the promising *Leiurus quinquestriatus* scorpion. Novel isolation techniques aim to deliver new molecules from SVs with neglectable cytotoxic effects by chemical manipulation of the target peptide Expanding the pool of SV sources still present a challenge since only a small volume of venom is collected from scorpions. Apply novel techniques in drug synthesis could pave the way to a larger scale of manufacturing anti-cancer peptides with the high SV characteristic selectivity and specificity to cancer cells.

## Data Availability

All data presented in this review are available and present in the text.

## Abbreviations

a.a.: Amino Acids
A549: Human lung adenocarcinoma
Aa: *Androctonus aeneas* Scorpion
Ama: *Androctonus **mauritanicus* Scorpion
BC: Breast Cancer
Bcl-2: B-cell lymphoma 2
Bcl-xL: Basal cell lymphoma-extra large
BmHYA1: Purified hyaluronidase from Chinese red scorpion Buthus martensii
BmK: *Bu* *thus* *martensii karsch*
BmK AGAP: Neurotoxin specific to Na^+^-channels
C6: Rat glioma cells
CDK4: Cyclin dependent kinase 4
ChTx: Charybdotoxin
CTX or ClTx: Chlorotoxin/ or chloride-bound toxins (Cl^−^ channel scorpion toxins)
DNA: Deoxyribonucleic acid
EMT: Epithelial-mesenchymal transition
ERα: Estrogen receptor alpha
FAK: Focal adhesion kinase
H22: Hepatocellular carcinoma in mice
HCT116: Colorectal cancer cell line (human colon-cancer cell line)
HeLa: Immortalized cell line derived from cervical cancer cells
Hb: *Heterometrus bengalensis Koch*
Hl: *Heterometrus liangi*
I.p.: Interperitoneally
IbTx: Iberiotoxin
IC_50_: Half maximal inhibitory concentration,
ICD-85: Venom derived peptides
IL-2: Interleukin 2
IL-6: Interleukin 6
Im: *Isometrus masculatus*
Jurkat cells: Human T lymphoma
K562: Chronic myelogenous leukemia
KCa: Calcium-activated potassium channels
KCNMA 1: Calcium-activated potassium channel
KDa: Kilodalton
KTx: Potassium-bound toxins (K^+^ channel scorpion toxins)
KV: Kilovolt
Kv: Voltage-gated potassium channel
LDH: Lactate dehydrogenase
MAPK: Mitogen-activated proteins
MCF-7: Michigan cancer foundation-7 (breast cancer cells)
MDA-MB-231: Triple-negative breast cancer cells (epithelial human breast cancer cell line)
MgTx: Margatoxin
Mg/L: Milligram per liter
MIC: Minimal inhibitory concentration
MMP-2: Matrix metallopeptidase 2
MRC-5: Normal fibroblasts
NDBPs: Non-disulfide bridges peptides
NF-kβ: Nuclear factor-Kβ
nM: Nanometer
NSCLC: Non-small-cell lung cancer
Osk-1: *Orthochirus scrobiculosus* selective to K^+^
PANC-1: Pancreatic cancer cells
PC: Prostate cancer
PESV: Polypeptides Isolated from *Bu**thus** martensii kirsch* scorpion
PTX3: Pentraxin-3
ROS: Reactive oxygen species
S180: Sarcoma
SCLC: Small-cell lung cancer
SHG-44: Human glioma cells
SK-Mel: Melanoma
Smp: *Scorpio maurus palmatus*
SV: Scorpion Venom
SVCIII: Scorpion venom fraction III (Bmk fraction)
SVPs: Scorpion venom-derived peptides
SVs: Scorpion venoms
THP-I: Human acute monocytic leukemia cell line
TM601: Synthetical product of CTX
TMZ: Temozolomide
TNF-α: Tumor necrosis factor
TsV: *Tityus serrulatus* venom
U87-MG: Glioblastoma cell line
U937: Human leukemia cell (histiocytic lymphoma)
VGSCs: Voltage-gated sodium channels
Vm: Negative membrane potential
µm: Micrometre

## References

[CR1] Ahmadi S, Knerr JM, Argemi L, Bordon KCF, Pucca MB, Cerni FA, Arantes EC, Çalışkan F, Laustsen AH (2020) Scorpion venom: detriments and benefits. Biomedicines. 10.3390/biomedicines8050118 32408604 10.3390/biomedicines8050118 PMC7277529

[CR2] Al Asmari AK, Khan AQ (2016) Investigation of in vivo potential of scorpion venom against skin tumorigenesis in mice via targeting markers associated with cancer development. Drug Des Devel Ther 10:3387–3397. 10.2147/dddt.S113171 27799739 10.2147/DDDT.S113171 PMC5076799

[CR3] Al-Asmari AK, Islam M, Al-Zahrani AM (2016a) In vitro analysis of the anticancer properties of scorpion venom in colorectal and breast cancer cell lines. Oncol Lett 11(2):1256–1262. 10.3892/ol.2015.4036 26893728 10.3892/ol.2015.4036 PMC4734223

[CR4] Al-Asmari AK, Riyasdeen A, Abbasmanthiri R, Arshaduddin M, Al-Harthi FA (2016b) Scorpion (*Androctonus bicolor*) venom exhibits cytotoxicity and induces cell cycle arrest and apoptosis in breast and colorectal cancer cell lines. Indian J Pharmacol 48(5):537–543. 10.4103/0253-7613.190742 27721540 10.4103/0253-7613.190742 PMC5051248

[CR5] Al-Asmari AK, Riyasdeen A, Islam M (2018a) Scorpion venom causes apoptosis by increasing reactive oxygen species and cell cycle arrest in MDA-MB-231 and HCT-8 cancer cell lines. J Evid Based Integr Med 23:2156587217751796. 10.1177/2156587217751796 29405760 10.1177/2156587217751796 PMC5881405

[CR6] Al-Asmari AK, Riyasdeen A, Islam M (2018b) Scorpion venom causes upregulation of p53 and downregulation of Bcl-x(L) and BID protein expression by modulating signaling proteins Erk(1/2) and STAT3, and DNA damage in breast and colorectal cancer cell lines. Integr Cancer Ther 17(2):271–281. 10.1177/1534735417704949 28438053 10.1177/1534735417704949 PMC6041906

[CR7] Almaaytah A, Albalas Q (2014) Scorpion venom peptides with no disulfide bridges: a review. Peptides 51:35–45. 10.1016/j.peptides.2013.10.021 24184590 10.1016/j.peptides.2013.10.021

[CR8] Almeida FM, Pimenta AM, De Figueiredo SG, Santoro MM, Martin-Eauclaire MF, Diniz CR, De Lima ME (2002) Enzymes with gelatinolytic activity can be found in *Tityus bahiensis* and *Tityus serrulatus* venoms. Toxicon 40(7):1041–1045. 10.1016/s0041-0101(02)00084-3 12076659 10.1016/s0041-0101(02)00084-3

[CR9] Arnold M, Morgan E, Rumgay H, Mafra A, Singh D, Laversanne M, Vignat J, Gralow JR, Cardoso F, Siesling S (2022) Current and future burden of breast cancer: global statistics for 2020 and 2040. The Breast 66:15–23 36084384 10.1016/j.breast.2022.08.010 PMC9465273

[CR10] Aydar E, Yeo S, Djamgoz M, Palmer C (2009) Abnormal expression, localization and interaction of canonical transient receptor potential ion channels in human breast cancer cell lines and tissues: a potential target for breast cancer diagnosis and therapy. Cancer Cell Int 9:23. 10.1186/1475-2867-9-23 19689790 10.1186/1475-2867-9-23 PMC2737535

[CR11] Bernardes-Oliveira E, Farias KJS, Gomes DL, de Araújo JMG, da Silva WD, Rocha HAO, Donadi EA, Fernandes-Pedrosa MF, Crispim JCO (2019) *Tityus serrulatus* scorpion venom induces apoptosis in cervical cancer cell lines. Evid Based Complement Alternat Med 2019:5131042. 10.1155/2019/5131042 31341494 10.1155/2019/5131042 PMC6612397

[CR12] Boltman T, Meyer M, Ekpo O (2023) Diagnostic and therapeutic approaches for glioblastoma and neuroblastoma cancers using chlorotoxin nanoparticles. Cancers. 10.3390/cancers15133388 37444498 10.3390/cancers15133388 PMC10341066

[CR13] Bordon KCF, Cologna CT, Fornari-Baldo EC, Pinheiro-Júnior EL, Cerni FA, Amorim FG, Anjolette FAP, Cordeiro FA, Wiezel GA, Cardoso IA, Ferreira IG, de Oliveira IS, Boldrini-França J, Pucca MB, Baldo MA, Arantes EC (2020) From animal poisons and venoms to medicines: achievements, challenges and perspectives in drug discovery. Front Pharmacol 11:1132. 10.3389/fphar.2020.01132 32848750 10.3389/fphar.2020.01132 PMC7396678

[CR14] Brackenbury WJ (2012) Voltage-gated sodium channels and metastatic disease. Channels (Austin) 6(5):352–361. 10.4161/chan.21910 22992466 10.4161/chan.21910 PMC3508774

[CR15] Brisson L, Driffort V, Benoist L, Poet M, Counillon L, Antelmi E, Rubino R, Besson P, Labbal F, Chevalier S, Reshkin SJ, Gore J, Roger S (2013) NaV15 Na⁺ channels allosterically regulate the NHE-1 exchanger and promote the activity of breast cancer cell invadopodia. J Cell Sci 126(Pt21):4835–4842. 10.1242/jcs.123901 23902689 10.1242/jcs.123901

[CR16] Bustin SA, Li SR, Dorudi S (2001) Expression of the Ca2+-activated chloride channel genes CLCA1 and CLCA2 is downregulated in human colorectal cancer. DNA Cell Biol 20(6):331–338. 10.1089/10445490152122442 11445004 10.1089/10445490152122442

[CR17] Capatina AL, Lagos D, Brackenbury WJ (2022) Targeting ion channels for cancer treatment: current progress and future challenges. Rev Physiol Biochem Pharmacol 183:1–43. 10.1007/112_2020_46 32865696 10.1007/112_2020_46

[CR18] Carmona G, Perera U, Gillett C, Naba A, Law AL, Sharma VP, Wang J, Wyckoff J, Balsamo M, Mosis F, De Piano M, Monypenny J, Woodman N, McConnell RE, Mouneimne G, Van Hemelrijck M, Cao Y, Condeelis J, Hynes RO, Gertler FB, Krause M (2016) Lamellipodin promotes invasive 3D cancer cell migration via regulated interactions with Ena/VASP and SCAR/WAVE. Oncogene 35(39):5155–5169. 10.1038/onc.2016.47 26996666 10.1038/onc.2016.47 PMC5031503

[CR19] Chen Y, Deng Y, Zhu C, Xiang C (2020) Anti prostate cancer therapy: aptamer-functionalized, curcumin and cabazitaxel co-delivered, tumor targeted lipid-polymer hybrid nanoparticles. Biomed Pharmacother 127:110181. 10.1016/j.biopha.2020.110181 32416561 10.1016/j.biopha.2020.110181

[CR20] Chen Y, Xu E, Sang M, Wang Z, Zhang Y, Ye J, Zhou Q, Zhao C, Hu C, Lu W, Cao P (2022) Makatoxin-3, a thermostable Nav1.7 agonist from *Buthus martensii Karsch* (BmK) scorpion elicits non-narcotic 99analgesia in inflammatory pain models. J Ethnopharmacol 288:114998. 10.1016/j.jep.2022.114998 35063590 10.1016/j.jep.2022.114998

[CR21] Chung S, Sugimoto Y, Huang J, Zhang M (2023) Iron oxide nanoparticles decorated with functional peptides for a targeted siRNA delivery to glioma cells. ACS Appl Mater Interfaces 15(1):106–119. 10.1021/acsami.2c17802 36442077 10.1021/acsami.2c17802 PMC11495154

[CR22] Cloudsley-Thompson JL (1993). Scorpions in mythology, folklore, and history. BOll. ACC. GIOENIA SCI. NAT. 26(345):53–63

[CR23] Comes N, Bielanska J, Vallejo-Gracia A, Serrano-Albarrás A, Marruecos L, Gómez D, Soler C, Condom E, Ramón YCS, Hernández-Losa J, Ferreres JC, Felipe A (2013) The voltage-dependent K(+) channels Kv1.3 and Kv1.5 in human cancer. Front Physiol 4:283. 10.3389/fphys.2013.00283 24133455 10.3389/fphys.2013.00283 PMC3794381

[CR24] Comes N, Serrano-Albarrás A, Capera J, Serrano-Novillo C, Condom E, Ramón YCS, Ferreres JC, Felipe A (2015) Involvement of potassium channels in the progression of cancer to a more malignant phenotype. Biochim Biophys Acta 1848(10PtB):2477–2492. 10.1016/j.bbamem.2014.12.008 25517985 10.1016/j.bbamem.2014.12.008

[CR25] Cordeiro FA, Amorim FG, Anjolette FAP, Arantes EC (2015) Arachnids of medical importance in Brazil: main active compounds present in scorpion and spider venoms and tick saliva. J Venom Anim Toxins Incl Trop Diseas 21(00):00–00 10.1186/s40409-015-0028-5 PMC4535291 26273285

[CR26] Cupo P (2015) Clinical update on scorpion envenoming. Rev Soc Bras Med Trop 48(6):642–649. 10.1590/0037-8682-0237-2015 26676487 10.1590/0037-8682-0237-2015

[CR27] Dardevet L, Rani D, Aziz TA, Bazin I, Sabatier JM, Fadl M, Brambilla E, De Waard M (2015) Chlorotoxin: a helpful natural scorpion peptide to diagnose glioma and fight tumor invasion. Toxins (Basel) 7(4):1079–1101. 10.3390/toxins7041079 25826056 10.3390/toxins7041079 PMC4417956

[CR28] DeBin JA, Maggio JE, Strichartz GR (1993) Purification and characterization of chlorotoxin, a chloride channel ligand from the venom of the scorpion. Am J Physiol 264(2 Pt 1):C361-369. 10.1152/ajpcell.1993.264.2.C361 8383429 10.1152/ajpcell.1993.264.2.C361

[CR29] Desales-Salazar E, Khusro A, Cipriano-Salazar M, Barbabosa-Pliego A, Rivas-Caceres RR (2020) Scorpion venoms and associated toxins as anticancer agents: update on their application and mechanism of action. J Appl Toxicol 40(10):1310–1324. 10.1002/jat.3976 32249452 10.1002/jat.3976

[CR30] Deshane J, Garner CC, Sontheimer H (2003) Chlorotoxin inhibits glioma cell invasion via matrix metalloproteinase-2. J Biol Chem 278(6):4135–4144. 10.1074/jbc.M205662200 12454020 10.1074/jbc.M205662200

[CR31] Ding J, Chua PJ, Bay BH, Gopalakrishnakone P (2014) Scorpion venoms as a potential source of novel cancer therapeutic compounds. Exp Biol Med 239(4):387–393. 10.1177/1535370213513991 10.1177/1535370213513991 24599885

[CR32] D’Suze G, Rosales A, Salazar V, Sevcik C (2010) Apoptogenic peptides from *Tityus discrepans* scorpion venom acting against the SKBR3 breast cancer cell line. Toxicon 56(8):1497–1505. 10.1016/j.toxicon.2010.09.008 20888852 10.1016/j.toxicon.2010.09.008

[CR33] Du Q, Hou X, Wang L, Zhang Y, Xi X, Wang H, Zhou M, Duan J, Wei M, Chen T (2015) AaeAP1 and AaeAP2: novel antimicrobial peptides from the venom of the scorpion, *Androctonus aeneas*: structural characterisation, molecular cloning of biosynthetic precursor-encoding cDNAs and engineering of analogues with enhanced antimicrobial and anticancer activities. Toxins 7(2):219–237 25626077 10.3390/toxins7020219 PMC4344621

[CR34] Dueñas-Cuellar RA, Kushmerick C, Naves LA, Batista IF, Guerrero-Vargas JA, Pires OR Jr, Fontes W, Castro MS (2015) Cm38: a new antimicrobial peptide active against Klebsiella pneumoniae is homologous to Cn11. Protein Pept Lett 22(2):164–172. 10.2174/092986652202150128143048 25633390 10.2174/092986652202150128143048

[CR35] Dueñas-Cuellar RA, Santana CJC, Magalhães ACM, Pires OR Jr, Fontes W, Castro MS (2020) Scorpion toxins and ion channels: potential applications in cancer therapy. Toxins. 10.3390/toxins12050326 32429050 10.3390/toxins12050326 PMC7290751

[CR36] El-Ghlban S, Kasai T, Shigehiro T, Yin HX, Sekhar S, Ida M, Sanchez A, Mizutani A, Kudoh T, Murakami H, Seno M (2014) Chlorotoxin-Fc fusion inhibits release of MMP-2 from pancreatic cancer cells. Biomed Res Int 2014:152659. 10.1155/2014/152659 10.1155/2014/152659 PMC3910484 24511528

[CR37] Escárcega RO, Fuentes-Alexandro S, García-Carrasco M, Gatica A, Zamora A (2007) The transcription factor nuclear factor-kappa B and cancer. Clin Oncol (r Coll Radiol) 19(2):154–161. 10.1016/j.clon.2006.11.013 17355113 10.1016/j.clon.2006.11.013

[CR38] Feng L, Gao R, Gopalakrishnakone P (2008) Isolation and characterization of a hyaluronidase from the venom of Chinese red scorpion *Buthus martensi*. Comp Biochem Physiol C Toxicol Pharmacol 148(3):250–257. 10.1016/j.cbpc.2008.06.003 18611448 10.1016/j.cbpc.2008.06.003

[CR39] Feske S, Wulff H, Skolnik EY (2015) Ion channels in innate and adaptive immunity. Annu Rev Immunol 33:291–353. 10.1146/annurev-immunol-032414-112212 25861976 10.1146/annurev-immunol-032414-112212 PMC4822408

[CR40] Fu YJ, Yin LT, Liang AH, Zhang CF, Wang W, Chai BF, Yang JY, Fan XJ (2007) Therapeutic potential of chlorotoxin-like neurotoxin from the Chinese scorpion for human gliomas. Neurosci Lett 412(1):62–67. 10.1016/j.neulet.2006.10.056 17166663 10.1016/j.neulet.2006.10.056

[CR41] Fu YJ, An N, Chan KG, Wu YB, Zheng SH, Liang AH (2011) A model of BmK CT in inhibiting glioma cell migration via matrix metalloproteinase-2 from experimental and molecular dynamics simulation study. Biotechnol Lett 33(7):1309–1317. 10.1007/s10529-011-0587-7 21424168 10.1007/s10529-011-0587-7

[CR42] Fu Y, An N, Li K, Zheng Y, Liang A (2012) Chlorotoxin-conjugated nanoparticles as potential glioma-targeted drugs. J Neurooncol 107(3):457–462. 10.1007/s11060-011-0763-6 22108716 10.1007/s11060-011-0763-6

[CR43] Gao B, Xu J, Rodriguez Mdel C, Lanz-Mendoza H, Hernández-Rivas R, Du W, Zhu S (2010) Characterization of two linear cationic antimalarial peptides in the scorpion *Mesobuthus eupeus*. Biochimie 92(4):350–359. 10.1016/j.biochi.2010.01.011 20097251 10.1016/j.biochi.2010.01.011

[CR44] Giangiacomo KM, Ceralde Y, Mullmann TJ (2004) Molecular basis of alpha-KTx specificity. Toxicon 43(8):877–886. 10.1016/j.toxicon.2003.11.029 15208020 10.1016/j.toxicon.2003.11.029

[CR45] Goudet C, Chi CW, Tytgat J (2002) An overview of toxins and genes from the venom of the Asian scorpion *Buthus martensi Karsch*. Toxicon 40(9):1239–1258. 10.1016/s0041-0101(02)00142-3 12220709 10.1016/s0041-0101(02)00142-3

[CR46] Griffin M, Khan R, Basu S, Smith S (2020) Ion channels as therapeutic targets in high grade gliomas. Cancers. 10.3390/cancers12103068 33096667 10.3390/cancers12103068 PMC7589494

[CR47] Gubič Š, Hendrickx LA, Toplak Ž, Sterle M, Peigneur S, Tomašič T, Pardo LA, Tytgat J, Zega A, Mašič LP (2021) Discovery of K(V) 1.3 ion channel inhibitors: Medicinal chemistry approaches and challenges. Med Res Rev 41(4):2423–2473. 10.1002/med.21800 33932253 10.1002/med.21800 PMC8252768

[CR48] Guilhelmelli F, Vilela N, Smidt KS, de Oliveira MA, da Cunha Morales Álvares A, Rigonatto MC, da Silva Costa PH, Tavares AH, de Freitas SM, Nicola AM, Franco OL, Derengowski LD, Schwartz EF, Mortari MR, Bocca AL, Albuquerque P, Silva-Pereira I (2016) Activity of scorpion venom-derived antifungal peptides against planktonic cells of *Candida spp.* and *Cryptococcus neoformans* and *Candida** albicans* biofilms. Front Microbiol 7:1844. 10.3389/fmicb.2016.01844 27917162 10.3389/fmicb.2016.01844 PMC5114273

[CR49] Guo X, Ma C, Du Q, Wei R, Wang L, Zhou M, Chen T, Shaw C (2013) Two peptides, TsAP-1 and TsAP-2, from the venom of the Brazilian yellow scorpion, *Tityus serrulatus:* evaluation of their antimicrobial and anticancer activities. Biochimie 95(9):1784–1794. 10.1016/j.biochi.2013.06.003 23770440 10.1016/j.biochi.2013.06.003

[CR50] Gupta SD, Gomes A, Debnath A, Saha A, Gomes A (2010) Apoptosis induction in human leukemic cells by a novel protein Bengalin, isolated from Indian black scorpion venom: through mitochondrial pathway and inhibition of heat shock proteins. Chem Biol Interact 183(2):293–303. 10.1016/j.cbi.2009.11.006 19913524 10.1016/j.cbi.2009.11.006

[CR51] Han X, Wang F, Yao W, Xing H, Weng D, Song X, Chen G, Xi L, Zhu T, Zhou J, Xu G, Wang S, Meng L, Iadecola C, Wang G, Ma D (2007) Heat shock proteins and p53 play a critical role in K+ channel-mediated tumor cell proliferation and apoptosis. Apoptosis 12(10):1837–1846. 10.1007/s10495-007-0101-9 17624594 10.1007/s10495-007-0101-9

[CR52] Harrison PL, Abdel-Rahman MA, Strong PN, Tawfik MM, Miller K (2016) Characterisation of three alpha-helical antimicrobial peptides from the venom of scorpio maurus palmatus. Toxicon 117:30–36. 10.1016/j.toxicon.2016.03.014 27019370 10.1016/j.toxicon.2016.03.014

[CR53] Hayden MS, West AP, Ghosh S (2006) NF-kappaB and the immune response. Oncogene 25(51):6758–6780. 10.1038/sj.onc.1209943 17072327 10.1038/sj.onc.1209943

[CR54] Heather JM, Chain B (2016) The sequence of sequencers: the history of sequencing DNA. Genomics 107(1):1–8. 10.1016/j.ygeno.2015.11.003 26554401 10.1016/j.ygeno.2015.11.003 PMC4727787

[CR55] Heinen TE, da Veiga AB (2011) Arthropod venoms and cancer. Toxicon 57(4):497–511. 10.1016/j.toxicon.2011.01.002 21236287 10.1016/j.toxicon.2011.01.002

[CR56] Helms LM, Felix JP, Bugianesi RM, Garcia ML, Stevens S, Leonard RJ, Knaus HG, Koch R, Wanner SG, Kaczorowski GJ, Slaughter RS (1997) Margatoxin binds to a homomultimer of K(V)1.3 channels in Jurkat cells. Comparison with K(V)1.3 expressed in CHO cells. Biochemistry 36(12):3737–3744. 10.1021/bi962351p 9132027 10.1021/bi962351p

[CR57] Hmed B, Serria HT, Mounir ZK (2013) Scorpion peptides: potential use for new drug development. J Toxicol 2013:958797. 10.1155/2013/958797 23843786 10.1155/2013/958797 PMC3697785

[CR58] Huang J, Han S, Sun Q, Zhao Y, Liu J, Yuan X, Mao W, Peng B, Liu W, Yin J, He X (2017) Kv1.3 channel blocker (ImKTx88) maintains blood-brain barrier in experimental autoimmune encephalomyelitis. Cell Biosci 7:31. 10.1186/s13578-017-0158-2 28596825 10.1186/s13578-017-0158-2 PMC5463463

[CR59] Inceoglu B, Lango J, Rabinovich A, Whetstone P, Hammock BD (2006) The neutralizing effect of a polyclonal antibody raised against the n-terminal eighteen-aminoacid residues of birtoxin towards the whole venom of Parabuthus transvaalicus. Toxicon 47(2):144–149. 10.1016/j.toxicon.2005.08.018 16356521 10.1016/j.toxicon.2005.08.018

[CR60] Jacobs BA, Chetty A, Horsnell WGC, Schäfer G, Prince S, Smith KA (2018) Hookworm exposure decreases human papillomavirus uptake and cervical cancer cell migration through systemic regulation of epithelial-mesenchymal transition marker expression. Sci Rep 8(1):11547. 10.1038/s41598-018-30058-9 30069018 10.1038/s41598-018-30058-9 PMC6070561

[CR61] Jacoby DB, Dyskin E, Yalcin M, Kesavan K, Dahlberg W, Ratliff J, Johnson EW, Mousa SA (2010) Potent pleiotropic anti-angiogenic effects of TM601, a synthetic chlorotoxin peptide. Anticancer Res 30(1):39–46 20150615

[CR62] Jang SH, Choi SY, Ryu PD, Lee SY (2011) Anti-proliferative effect of Kv1.3 blockers in A549 human lung adenocarcinoma in vitro and in vivo. Eur J Pharmacol 651(1–3):26–32. 10.1016/j.ejphar.2010.10.066 21087602 10.1016/j.ejphar.2010.10.066

[CR63] de Jesus Oliveira T, Oliveira UC, da Silva Junior PI (2019) Serrulin: A glycine-rich bioactive peptide from the Hemolymph of the yellow *Tityus serrulatus* scorpion. Toxins (Basel). 10.3390/toxins11090517 31489876 10.3390/toxins11090517 PMC6784228

[CR64] Jia Z, Zhu X, Zhou Y, Wu J, Cao M, Hu C, Yu L, Xu R, Chen Z (2024) Polypeptides from traditional Chinese medicine: comprehensive review of perspective towards cancer management. Int J Biol Macromol. 10.1016/j.ijbiomac.2024.129423 38232868 10.1016/j.ijbiomac.2024.129423

[CR65] Jian D (2014) Screening and evaluation of the anticancer potential of scorpion venoms and snake venom l-amino acid oxidase in gastric cancer. https://scholarbank.nus.edu.sg/handle/10635/119451

[CR66] Jiapaer S, Furuta T, Tanaka S, Kitabayashi T, Nakada M (2018) Potential strategies overcoming the temozolomide resistance for glioblastoma. Neurol Med Chir 58(10):405–421. 10.2176/nmc.ra.2018-0141 10.2176/nmc.ra.2018-0141 PMC6186761 30249919

[CR67] Kampo S, Ahmmed B, Zhou T, Owusu L, Anabah TW, Doudou NR, Kuugbee ED, Cui Y, Lu Z, Yan Q, Wen QP (2019) Scorpion venom analgesic peptide, BmK AGAP inhibits stemness, and epithelial-mesenchymal transition by down-regulating PTX3 in breast cancer. Front Oncol 9:21. 10.3389/fonc.2019.00021 30740360 10.3389/fonc.2019.00021 PMC6355678

[CR68] Kheirandish Zarandi P, Zare Mirakabadi A, Sotoodehnejadnematalahi F (2019) Cytotoxic and anticancer effects of ICD-85 (venom derived peptides) in human breast adenocarcinoma and normal human dermal fibroblasts. Iran J Pharm Res 18(1):232–240 31089358 PMC6487428

[CR69] Krawczyk A, Arndt MA, Grosse-Hovest L, Weichert W, Giebel B, Dittmer U, Hengel H, Jäger D, Schneweis KE, Eis-Hübinger AM, Roggendorf M, Krauss J (2013) Overcoming drug-resistant herpes simplex virus (HSV) infection by a humanized antibody. Proc Natl Acad Sci U S A 110(17):6760–6765. 10.1073/pnas.1220019110 23569258 10.1073/pnas.1220019110 PMC3637765

[CR70] Lee WH, Loo CY, Ghadiri M, Leong CR, Young PM, Traini D (2018) The potential to treat lung cancer via inhalation of repurposed drugs. Adv Drug Deliv Rev 133:107–130. 10.1016/j.addr.2018.08.012 30189271 10.1016/j.addr.2018.08.012

[CR71] Li W, Xin Y, Chen Y, Li X, Zhang C, Bai J, Yuan J (2014) The anti-proliferative effects and mechanisms of low molecular weight scorpion BmK venom peptides on human hepatoma and cervical carcinoma cells in vitro. Oncol Lett 8(4):1581–1584. 10.3892/ol.2014.2336 25202371 10.3892/ol.2014.2336 PMC4156272

[CR72] Li B, Lyu P, Xi X, Ge L, Mahadevappa R, Shaw C, Kwok HF (2018) Triggering of cancer cell cycle arrest by a novel scorpion venom-derived peptide-gonearrestide. J Cell Mol Med 22(9):4460–4473. 10.1111/jcmm.13745 29993185 10.1111/jcmm.13745 PMC6111814

[CR73] Li Z, Hu P, Wu W, Wang Y (2019) Peptides with therapeutic potential in the venom of the scorpion *Buthus martensii Karsch*. Peptides 115:43–50. 10.1016/j.peptides.2019.02.009 30858089 10.1016/j.peptides.2019.02.009

[CR74] Li J, Zeng H, You Y, Wang R, Tan T, Wang W, Yin L, Zeng Z, Zeng Y, Xie T (2021) Active targeting of orthotopic glioma using biomimetic liposomes co-loaded elemene and cabazitaxel modified by transferritin. J Nanobiotechnology 19(1):289. 10.1186/s12951-021-01048-3 34565383 10.1186/s12951-021-01048-3 PMC8474941

[CR75] Ling C, Zhang Y, Li J, Chen W, Ling C (2019) Clinical use of toxic proteins and peptides from tian hua fen and scorpion venom. Curr Protein Pept Sci 20(3):285–295. 10.2174/1389203719666180622100641 29932034 10.2174/1389203719666180622100641

[CR76] Lippens G, Najib J, Wodak SJ, Tartar A (1995) NMR sequential assignments and solution structure of chlorotoxin, a small scorpion toxin that blocks chloride channels. Biochemistry 34(1):13–21. 10.1021/bi00001a003 7819188 10.1021/bi00001a003

[CR77] Liscano Y, Oñate-Garzón J, Delgado JP (2020) Peptides with dual antimicrobial-anticancer activity: strategies to overcome peptide limitations and rational design of anticancer peptides. Molecules. 10.3390/molecules25184245 32947811 10.3390/molecules25184245 PMC7570524

[CR78] Liu YF, Hu J, Zhang JH, Wang SL, Wu CF (2002) Isolation, purification, and n-terminal partial sequence of an antitumor peptide from the venom of the Chinese scorpion *Buthus martensii Karsch*. Prep Biochem Biotechnol 32(4):317–327. 10.1081/pb-120015456 12455825 10.1081/PB-120015456

[CR79] Luna-Ramirez K, Tonk M, Rahnamaeian M, Vilcinskas A (2017) Bioactivity of natural and engineered antimicrobial peptides from venom of the scorpions Urodacus yaschenkoi and U. manicatus. Toxins. 10.3390/toxins9010022 28067810 10.3390/toxins9010022 PMC5308254

[CR80] Lyons SA, O’Neal J, Sontheimer H (2002) Chlorotoxin, a scorpion-derived peptide, specifically binds to gliomas and tumors of neuroectodermal origin. Glia 39(2):162–173. 10.1002/glia.10083 12112367 10.1002/glia.10083

[CR81] Machado RJ, Estrela AB, Nascimento AK, Melo MM, Torres-Rêgo M, Lima EO, Rocha HA, Carvalho E, Silva-Junior AA, Fernandes-Pedrosa MF (2016) Characterization of TistH, a multifunctional peptide from the scorpion *Tityus stigmurus*: structure, cytotoxicity and antimicrobial activity. Toxicon 119:362–370. 10.1016/j.toxicon.2016.06.002 27267248 10.1016/j.toxicon.2016.06.002

[CR82] McFerrin MB, Sontheimer H (2006) A role for ion channels in glioma cell invasion. Neuron Glia Biol 2(1):39–49. 10.1017/s17440925x06000044 16520829 10.1017/S17440925X06000044 PMC1389710

[CR83] de Melo ET, Estrela AB, Santos EC, Machado PR, Farias KJ, Torres TM, Carvalho E, Lima JP, Silva-Júnior AA, Barbosa EG, Fernandes-Pedrosa Mde F (2015) Structural characterization of a novel peptide with antimicrobial activity from the venom gland of the scorpion Tityus stigmurus: Stigmurin. Peptides 68:3–10. 10.1016/j.peptides.2015.03.003 25805002 10.1016/j.peptides.2015.03.003

[CR84] Mendes LC, Viana GMM, Nencioni ALA, Pimenta DC, Beraldo-Neto E (2023) Scorpion peptides and ion channels: an insightful review of mechanisms and drug development. Toxins. 10.3390/toxins15040238 37104176 10.3390/toxins15040238 PMC10145618

[CR85] Mikaelian AG, Traboulay E, Zhang XM, Yeritsyan E, Pedersen PL, Ko YH, Matalka KZ (2020) Pleiotropic anticancer properties of scorpion venom peptides: rhopalurus princeps venom as an anticancer agent. Drug Des Devel Ther 14:881–893. 10.2147/dddt.S231008 32161447 10.2147/DDDT.S231008 PMC7051175

[CR86] Minniti G, Niyazi M, Alongi F, Navarria P, Belka C (2021) Current status and recent advances in reirradiation of glioblastoma. Radiat Oncol 16(1):36. 10.1186/s13014-021-01767-9 33602305 10.1186/s13014-021-01767-9 PMC7890828

[CR87] Monge-Fuentes V, Gomes FM, Campos GA, Silva Jde C, Biolchi AM, Dos Anjos LC, Gonçalves JC, Lopes KS, Mortari MR (2015) Neuroactive compounds obtained from arthropod venoms as new therapeutic platforms for the treatment of neurological disorders. J Venom Anim Toxin Incl Trop Dis 21:31. 10.1186/s40409-015-0031-x 10.1186/s40409-015-0031-x PMC4529710 26257776

[CR88] Northcott PA, Dubuc AM, Pfister S, Taylor MD (2012) Molecular subgroups of medulloblastoma. Expert Rev Neurother 12(7):871–884. 10.1586/ern.12.66 22853794 10.1586/ern.12.66 PMC4334443

[CR89] Oeggerli M, Tian Y, Ruiz C, Wijker B, Sauter G, Obermann E, Güth U, Zlobec I, Sausbier M, Kunzelmann K, Bubendorf L (2012) Role of KCNMA1 in breast cancer. PLoS ONE 7(8):e41664. 10.1371/journal.pone.0041664 22899999 10.1371/journal.pone.0041664 PMC3416802

[CR90] Ortiz E, Gurrola GB, Schwartz EF, Possani LD (2015) Scorpion venom components as potential candidates for drug development. Toxicon 93:125–135. 10.1016/j.toxicon.2014.11.233 25432067 10.1016/j.toxicon.2014.11.233 PMC7130864

[CR91] Ouadid-Ahidouch H, Roudbaraki M, Ahidouch A, Delcourt P, Prevarskaya N (2004) Cell-cycle-dependent expression of the large Ca2 ± activated K+ channels in breast cancer cells. Biochem Biophys Res Commun 316(1):244–251 15003537 10.1016/j.bbrc.2004.02.041

[CR92] Padilla-Rodriguez M, Parker SS, Adams DG, Westerling T, Puleo JI, Watson AW, Hill SM, Noon M, Gaudin R, Aaron J, Tong D, Roe DJ, Knudsen B, Mouneimne G (2018) The actin cytoskeletal architecture of estrogen receptor positive breast cancer cells suppresses invasion. Nat Commun 9(1):2980. 10.1038/s41467-018-05367-2 30061623 10.1038/s41467-018-05367-2 PMC6065369

[CR93] Padmanabhan M, Prince PS (2006) Preventive effect of S-allylcysteine on lipid peroxides and antioxidants in normal and isoproterenol-induced cardiotoxicity in rats: a histopathological study. Toxicology 224(1–2):128–137. 10.1016/j.tox.2006.04.039 16757080 10.1016/j.tox.2006.04.039

[CR94] Parente AMS, Daniele-Silva A, Furtado AA, Melo MA, Lacerda AF, Queiroz M, Moreno C, Santos E, Rocha HAO, Barbosa EG, Carvalho E, Silva-Júnior AA, Silva MS, Fernandes-Pedrosa MF (2018) Analogs of the scorpion venom peptide Stigmurin: structural assessment, toxicity, and increased antimicrobial activity. Toxins. 10.3390/toxins10040161 29670004 10.3390/toxins10040161 PMC5923327

[CR95] Pedersen SF, Stock C (2013) Ion channels and transporters in cancer: pathophysiology, regulation, and clinical potential. Cancer Res 73(6):1658–1661. 10.1158/0008-5472.Can-12-4188 23302229 10.1158/0008-5472.CAN-12-4188

[CR96] Pedron CN, Torres MT, Lima J, Silva PI, Silva FD, Oliveira VX (2017) Novel designed VmCT1 analogs with increased antimicrobial activity. Eur J Med Chem 126:456–463. 10.1016/j.ejmech.2016.11.040 27912176 10.1016/j.ejmech.2016.11.040

[CR97] Pessini AC, Takao TT, Cavalheiro EC, Vichnewski W, Sampaio SV, Giglio JR, Arantes EC (2001) A hyaluronidase from *Tityus serrulatus* scorpion venom: isolation, characterization and inhibition by flavonoids. Toxicon 39(10):1495–1504. 10.1016/s0041-0101(01)00122-2 11478957 10.1016/s0041-0101(01)00122-2

[CR98] Petricevich VL (2004) Cytokine and nitric oxide production following severe envenomation. Curr Drug Targets Inflamm Allergy 3(3):325–332. 10.2174/1568010043343642 15379602 10.2174/1568010043343642

[CR99] Possani LD, Merino E, Corona M, Bolivar F, Becerril B (2000) Peptides and genes coding for scorpion toxins that affect ion-channels. Biochimie 82(9–10):861–868 11086216 10.1016/s0300-9084(00)01167-6

[CR100] Pucca MB, Bertolini TB, Cerni FA, Bordon KC, Peigneur S, Tytgat J, Bonato VL, Arantes EC (2016) Immunosuppressive evidence of *Tityus serrulatus* toxins Ts6 and Ts15: insights of a novel K(+) channel pattern in T cells. Immunology 147(2):240–250. 10.1111/imm.12559 26595158 10.1111/imm.12559 PMC4717234

[CR101] Qin C, He B, Dai W, Zhang H, Wang X, Wang J, Zhang X, Wang G, Yin L, Zhang Q (2014) Inhibition of metastatic tumor growth and metastasis via targeting metastatic breast cancer by chlorotoxin-modified liposomes. Mol Pharm 11(10):3233–3241. 10.1021/mp400691z 24559485 10.1021/mp400691z

[CR102] Quintero-Hernández V, Jiménez-Vargas JM, Gurrola GB, Valdivia HH, Possani LD (2013) Scorpion venom components that affect ion-channels function. Toxicon 76:328–342 23891887 10.1016/j.toxicon.2013.07.012 PMC4089097

[CR103] Ramírez A, Vera E, Gamboa-Domínguez A, Lambert P, Gariglio P, Camacho J (2018) Calcium-activated potassium channels as potential early markers of human cervical cancer. Oncol Lett 15(5):7249–7254. 10.3892/ol.2018.8187 29725443 10.3892/ol.2018.8187 PMC5920501

[CR104] Rizzo A, Santoni M, Mollica V, Fiorentino M, Brandi G, Massari F (2022) Microbiota and prostate cancer. Semin Cancer Biol 86(Pt 3):1058–1065. 10.1016/j.semcancer.2021.09.007 34536504 10.1016/j.semcancer.2021.09.007

[CR105] Rjeibi I, Mabrouk K, Mosrati H, Berenguer C, Mejdoub H, Villard C, Laffitte D, Bertin D, Ouafik LH, Luis J, ElAyeb M, Srairi-Abid N (2011) Purification, synthesis and characterization of AaCtx, the first chlorotoxin-like peptide from Androctonus australis scorpion venom. Peptides 32(4):656–663. 10.1016/j.peptides.2011.01.015 21262299 10.1016/j.peptides.2011.01.015

[CR106] Romo Vaquero M, Yáñez-Gascón MJ, García Villalba R, Larrosa M, Fromentin E, Ibarra A, Roller M, Tomás-Barberán F, Espín de Gea JC, García-Conesa MT (2012) Inhibition of gastric lipase as a mechanism for body weight and plasma lipids reduction in Zucker rats fed a rosemary extract rich in carnosic acid. PLoS ONE 7(6):e39773. 10.1371/journal.pone.0039773 22745826 10.1371/journal.pone.0039773 PMC3382157

[CR107] Ruiming Z, Yibao M, Yawen H, Zhiyong D, Yingliang W, Zhijian C, Wenxin L (2010) Comparative venom gland transcriptome analysis of the scorpion Lychas mucronatus reveals intraspecific toxic gene diversity and new venomous components. BMC Genom 11:452. 10.1186/1471-2164-11-452 10.1186/1471-2164-11-452 PMC3091649 20663230

[CR108] Salem ML, Shoukry NM, Teleb WK, Abdel-Daim MM, Abdel-Rahman MA (2016) In vitro and in vivo antitumor effects of the Egyptian scorpion Androctonus amoreuxi venom in an Ehrlich ascites tumor model. Springerplus 5:570. 10.1186/s40064-016-2269-3 27247867 10.1186/s40064-016-2269-3 PMC4864766

[CR109] Santussi WM, Bordon KCF, Rodrigues Alves APN, Cologna CT, Said S, Arantes EC (2017) Antifungal activity against filamentous fungi of Ts1, a multifunctional toxin from *Tityus serrulatus *scorpion venom. Front Microbiol 8:984. 10.3389/fmicb.2017.00984 28634472 10.3389/fmicb.2017.00984 PMC5459920

[CR110] Sawaya RE, Yamamoto M, Gokaslan ZL, Wang SW, Mohanam S, Fuller GN, McCutcheon IE, Stetler-Stevenson WG, Nicolson GL, Rao JS (1996) Expression and localization of 72 kDa type IV collagenase (MMP-2) in human malignant gliomas in vivo. Clin Exp Metastasis 14(1):35–42. 10.1007/bf00157684 8521615 10.1007/BF00157684

[CR111] Shahzadi SK, Karuvantevida N, Banerjee Y (2021) A venomics approach to the identification and characterization of bioactive peptides from animal venoms for colorectal cancer therapy: protocol for a proof-of-concept study. JMIR Res Protoc 10(12):e31128. 10.2196/31128 34932002 10.2196/31128 PMC8734912

[CR112] Shao J-H, Cui Y, Zhao M-Y, Wu C-F, Liu Y-F, Zhang J-H (2014) Purification, characterization, and bioactivity of a new analgesic-antitumor peptide from Chinese scorpion *Buthus martensii Karsch*. Peptides 53:89–96. 10.1016/j.peptides.2013.10.023 24269605 10.1016/j.peptides.2013.10.023

[CR113] Simone Y, van der Meijden A (2021) Armed stem to stinger: a review of the ecological roles of scorpion weapons. J Venom Anim Toxins Incl Trop Dis 27:e20210002. 10.1590/1678-9199-jvatitd-2021-0002 34527038 10.1590/1678-9199-JVATITD-2021-0002 PMC8425188

[CR114] Soleglad ME, Fet V (2003) High-level systematics and phylogeny of the extant scorpions (scorpiones: orthosterni). Euscorpius 2003(11):1–56

[CR115] Song X, Zhang G, Sun A, Guo J, Tian Z, Wang H, Liu Y (2012) Scorpion venom component III inhibits cell proliferation by modulating NF-κB activation in human leukemia cells. Exp Ther Med 4(1):146–150. 10.3892/etm.2012.548 23060939 10.3892/etm.2012.548 PMC3460309

[CR116] Sontheimer H (2008) An unexpected role for ion channels in brain tumor metastasis. Exp Biol Med 233(7):779–791. 10.3181/0711-mr-308 10.3181/0711-MR-308 PMC2557067 18445774

[CR117] Soroceanu L, Gillespie Y, Khazaeli MB, Sontheimer H (1998) Use of chlorotoxin for targeting of primary brain tumors. Cancer Res 58(21):4871–4879 9809993

[CR118] Soroceanu L, Manning TJ Jr, Sontheimer H (1999) Modulation of glioma cell migration and invasion using Cl(–) and K(+) ion channel blockers. J Neurosci 19(14):5942–5954. 10.1523/jneurosci.19-14-05942.1999 10407033 10.1523/JNEUROSCI.19-14-05942.1999 PMC6783071

[CR119] Srairi-Abid N, Othman H, Aissaoui D, BenAissa R (2019) Anti-tumoral effect of scorpion peptides: Emerging new cellular targets and signaling pathways. Cell Calcium 80:160–174. 10.1016/j.ceca.2019.05.003 31108338 10.1016/j.ceca.2019.05.003

[CR120] Symeonidou I, Arsenopoulos K, Tzilves D, Soba B, Gabriël S, Papadopoulos E (2018) Human taeniasis/cysticercosis: a potentially emerging parasitic disease in Europe. Ann Gastroenterol 31(4):406–412. 10.20524/aog.2018.0260 29991885 10.20524/aog.2018.0260 PMC6033766

[CR121] Tewarie IA, Senders JT, Kremer S, Devi S, Gormley WB, Arnaout O, Smith TR, Broekman MLD (2021) Survival prediction of glioblastoma patients-are we there yet? A systematic review of prognostic modeling for glioblastoma and its clinical potential. Neurosurg Rev 44(4):2047–2057. 10.1007/s10143-020-01430-z 33156423 10.1007/s10143-020-01430-z PMC8338817

[CR122] Thakur N, Qureshi A, Kumar M (2012) AVPpred: collection and prediction of highly effective antiviral peptides. Nuc Acid Res. 10.1093/nar/gks450 10.1093/nar/gks450 PMC3394244 22638580

[CR123] Ullrich N, Sontheimer H (1996) Biophysical and pharmacological characterization of chloride currents in human astrocytoma cells. Am J Physiol 270(5Pt1):C1511-1521. 10.1152/ajpcell.1996.270.5.C1511 8967454 10.1152/ajpcell.1996.270.5.C1511

[CR124] Uzair B, Bint EIS, Khan BA, Azad B, Mahmood T, Rehman MU, Braga VA (2018) Scorpion venom peptides as a potential source for human drug candidates. Protein Pept Lett 25(7):702–708. 10.2174/0929866525666180614114307 29921194 10.2174/0929866525666180614114307

[CR125] Veiseh M, Gabikian P, Bahrami SB, Veiseh O, Zhang M, Hackman RC, Ravanpay AC, Stroud MR, Kusuma Y, Hansen SJ, Kwok D, Munoz NM, Sze RW, Grady WM, Greenberg NM, Ellenbogen RG, Olson JM (2007) Tumor paint: a chlorotoxin:Cy5.5 bioconjugate for intraoperative visualization of cancer foci. Cancer Res 67(14):6882–6888. 10.1158/0008-5472.Can-06-3948 17638899 10.1158/0008-5472.CAN-06-3948

[CR126] Wang Y, Li K, Han S, Tian YH, Hu PC, Xu XL, He YQ, Pan WT, Gao Y, Zhang Z, Zhang JW, Wei L (2019) Chlorotoxin targets ERα/VASP signaling pathway to combat breast cancer. Cancer Med 8(4):1679–1693. 10.1002/cam4.2019 30806044 10.1002/cam4.2019 PMC6488122

[CR127] Ward MJ, Ellsworth SA, Nystrom GS (2018) A global accounting of medically significant scorpions: epidemiology, major toxins, and comparative resources in harmless counterparts. Toxicon 151:137–155. 10.1016/j.toxicon.2018.07.007 30009779 10.1016/j.toxicon.2018.07.007

[CR128] Wu XS, Jian XC, Yin B, He ZJ (2010) Development of the research on the application of chlorotoxin in imaging diagnostics and targeted therapies for tumors. Chin J Cancer 29(6):626–630. 10.5732/cjc.009.10359 20507737 10.5732/cjc.009.10359

[CR129] Wu S, Ma K, Qiao WL, Zhao LZ, Liu CC, Guo LL, Xing Y, Zhu ML, Zhao JH (2018) Anti-metastatic effect of 131I-labeled *Buthus martensii Karsch chlorotoxin* in gliomas. Int J Mol Med 42(6):3386–3394. 10.3892/ijmm.2018.3905 30272348 10.3892/ijmm.2018.3905 PMC6202110

[CR130] Zargan J, Sajad M, Umar S, Naime M, Ali S, Khan HA (2011a) Scorpion (*Androctonus crassicauda*) venom limits growth of transformed cells (SH-SY5Y and MCF-7) by cytotoxicity and cell cycle arrest. Exp Mol Pathol 91(1):447–454. 10.1016/j.yexmp.2011.04.008 21536027 10.1016/j.yexmp.2011.04.008

[CR131] Zargan J, Sajad M, Umar S, Naime M, Ali S, Khan HA (2011b) Scorpion (*Odontobuthus doriae*) venom induces apoptosis and inhibits DNA synthesis in human neuroblastoma cells. Mol Cell Biochem 348(1–2):173–181. 10.1007/s11010-010-0652-x 21061047 10.1007/s11010-010-0652-x

[CR132] Zargan J, Umar S, Sajad M, Naime M, Ali S, Khan HA (2011c) Scorpion venom (*Odontobuthus doriae*) induces apoptosis by depolarization of mitochondria and reduces S-phase population in human breast cancer cells (MCF-7). Toxicol Vitro 25(8):1748–1756. 10.1016/j.tiv.2011.09.002 10.1016/j.tiv.2011.09.002 21945044

[CR133] Zerouti K, Khemili D, Laraba-Djebari F, Hammoudi-Triki D (2021) Nontoxic fraction of scorpion venom reduces bacterial growth and inflammatory response in a mouse model of infection. Toxin Rev 40(3):310–324. 10.1080/15569543.2019.1614064

[CR134] Zhang YY, Wu LC, Wang ZP, Wang ZX, Jia Q, Jiang GS, Zhang WD (2009) Anti-proliferation effect of polypeptide extracted from scorpion venom on human prostate cancer cells in vitro. J Clin Med Res 1(1):24–31. 10.4021/jocmr2009.01.1220 22505961 10.4021/jocmr2009.01.1220 PMC3318865

[CR135] Zhao Y, Cai X, Ye T, Huo J, Liu C, Zhang S, Cao P (2011) Analgesic-antitumor peptide inhibits proliferation and migration of SHG-44 human malignant glioma cells. J Cell Biochem 112(9):2424–2434. 10.1002/jcb.23166 21538480 10.1002/jcb.23166

[CR136] Zhao Y, Huang J, Yuan X, Peng B, Liu W, Han S, He X (2015) Toxins targeting the Kv1.3 channel: potential immunomodulators for autoimmune diseases. Toxins 7(5):1749–1764. 10.3390/toxins7051749 25996605 10.3390/toxins7051749 PMC4448172

[CR137] Zhou XH, Yang D, Zhang JH, Liu CM, Lei KJ (1989) Purification and n-terminal partial sequence of anti-epilepsy peptide from venom of the scorpion *Buthus martensii Karsch*. Biochem J 257(2):509–517. 10.1042/bj2570509 2930463 10.1042/bj2570509 PMC1135608

